# Prevalence of abnormal cardiovascular magnetic resonance findings in recovered patients from COVID-19: a systematic review and meta-analysis

**DOI:** 10.1186/s12968-021-00792-7

**Published:** 2021-09-03

**Authors:** Jin Young Kim, Kyunghwa Han, Young Joo Suh

**Affiliations:** 1 Department of Radiology, Dongsan Hospital, Keimyung University College of Medicine, Daegu, Korea; 2 Department of Radiology, Research Institute of Radiological Science, Severance Hospital, Yonsei University College of Medicine, 50–1 Yonsei-ro, Seodaemun-gu, Seoul, 03722 Korea

**Keywords:** Cardiac magnetic resonance imaging, Magnetic resonance imaging, Coronavirus disease 2019

## Abstract

**Background:**

The prevalence of abnormal cardiovascular magnetic resonance (CMR) findings in recovered coronavirus disease 2019 (COVID-19) patients is unclear. This study aimed to investigate the prevalence of abnormal CMR findings in recovered COVID-19 patients.

**Methods:**

A systematic literature search was performed to identify studies that report the prevalence of abnormal CMR findings in recovered COVID-19 patients. The number of patients with abnormal CMR findings and diagnosis of myocarditis on CMR (based on the Lake Louise criteria) and each abnormal CMR parameter were extracted. Subgroup analyses were performed according to patient characteristics (athletes vs. non-athletes and normal vs. undetermined cardiac enzyme levels). The pooled prevalence and 95% confidence interval (CI) of each CMR finding were calculated. Study heterogeneity was assessed, and meta-regression analysis was performed to investigate factors associated with heterogeneity.

**Results:**

In total, 890 patients from 16 studies were included in the analysis. The pooled prevalence of one or more abnormal CMR findings in recovered COVID-19 patients was 46.4% (95% CI 43.2%–49.7%). The pooled prevalence of myocarditis and late gadolinium enhancement (LGE) was 14.0% (95% CI 11.6%–16.8%) and 20.5% (95% CI 17.7%–23.6%), respectively. Further, heterogeneity was observed (I^2^ > 50%, p < 0.1). In the subgroup analysis, the pooled prevalence of abnormal CMR findings and myocarditis was higher in non-athletes than in athletes (62.5% vs. 17.1% and 23.9% vs. 2.5%, respectively). Similarly, the pooled prevalence of abnormal CMR findings and LGE was higher in the undetermined than in the normal cardiac enzyme level subgroup (59.4% vs. 35.9% and 45.5% vs. 8.3%, respectively). Being an athlete was a significant independent factor related to heterogeneity in multivariate meta-regression analysis (p < 0.05).

**Conclusions:**

Nearly half of recovered COVID-19 patients exhibited one or more abnormal CMR findings. Athletes and patients with normal cardiac enzyme levels showed a lower prevalence of abnormal CMR findings than non-athletes and patients with undetermined cardiac enzyme levels.

*Trial registration* The study protocol was registered in the PROSPERO database (registration number: CRD42020225234).

**Supplementary Information:**

The online version contains supplementary material available at 10.1186/s12968-021-00792-7.

## Background

The spread of coronavirus disease 2019 (COVID-19) was rapid, and COVID-19 was quickly designated as a pandemic since the first identified case in December 2019 in Wuhan, China [1]. As of July 7, 2021, more than 184 million people have been diagnosed with COVID-19 and nearly 4 million have died of the infection [2]. Although COVID-19 is primarily a respiratory disease, cardiovascular complications have been reported [3, 4] and are associated with higher mortality and risk of severe COVID-19 [5, 6]. Cardiac involvement in COVID-19 can manifest as myocarditis, heart failure, acute coronary syndrome, or arrhythmias [4, 7]. Among these, myocarditis has clinical significance because myocardial inflammation can result in permanent myocardial damage and contribute to the development of arrhythmia or chronic heart failure [7, 8].

Cardiovascular magnetic resonance (CMR) is used to diagnose cardiovascular complications of COVID-19, such as acute myocarditis, using the recently updated Lake Louise criteria [9]. Individual reports and one systematic review of CMR findings in COVID-19 patients have been published to date; however, most focused on patients in the active disease stage [10]. Notably, recent data indicated that the prevalence of abnormal CMR findings, such as myocardial edema and late gadolinium enhancement (LGE), in recovered COVID-19 patients is substantial [11–22]; however, their prevalence is highly variable. Although the clinical significance of abnormal CMR findings in recovered COVID-19 patients is not yet fully understood, determining the prevalence of such findings in certain subgroups of patients would benefit clinical decision-making. For example, the presence of myocardial scars after myocarditis can lead to sudden cardiac death, especially in athletes. Consequently, the prevalence of abnormal CMR findings in athletes who have recovered from COVID-19 affects their return to play [23–25].

Therefore, the purpose of this study was to investigate the prevalence of abnormal CMR findings in recovered COVID-19 patients through meta-analysis.

## Methods

Our methods followed the recommendations of the preferred reporting items for systematic reviews and meta-analyses statement [26], and the study protocol was registered in the PROSPERO database (registration number: CRD42020225234).

### Literature search

Two cardiothoracic radiologists with 5 and 8 years of experience, in performing meta-analyses designed the search strategy in consensus. Each individual independently performed systematic searches of PubMed, EMBASE, the Cochrane library, SSRN, and MedRxiv/BioRxiv on March 3, 2021, to identify studies published since 2020. The search terms are listed in Additional file 1: Appendix S1.

### Study selection

**Fig. 1:**
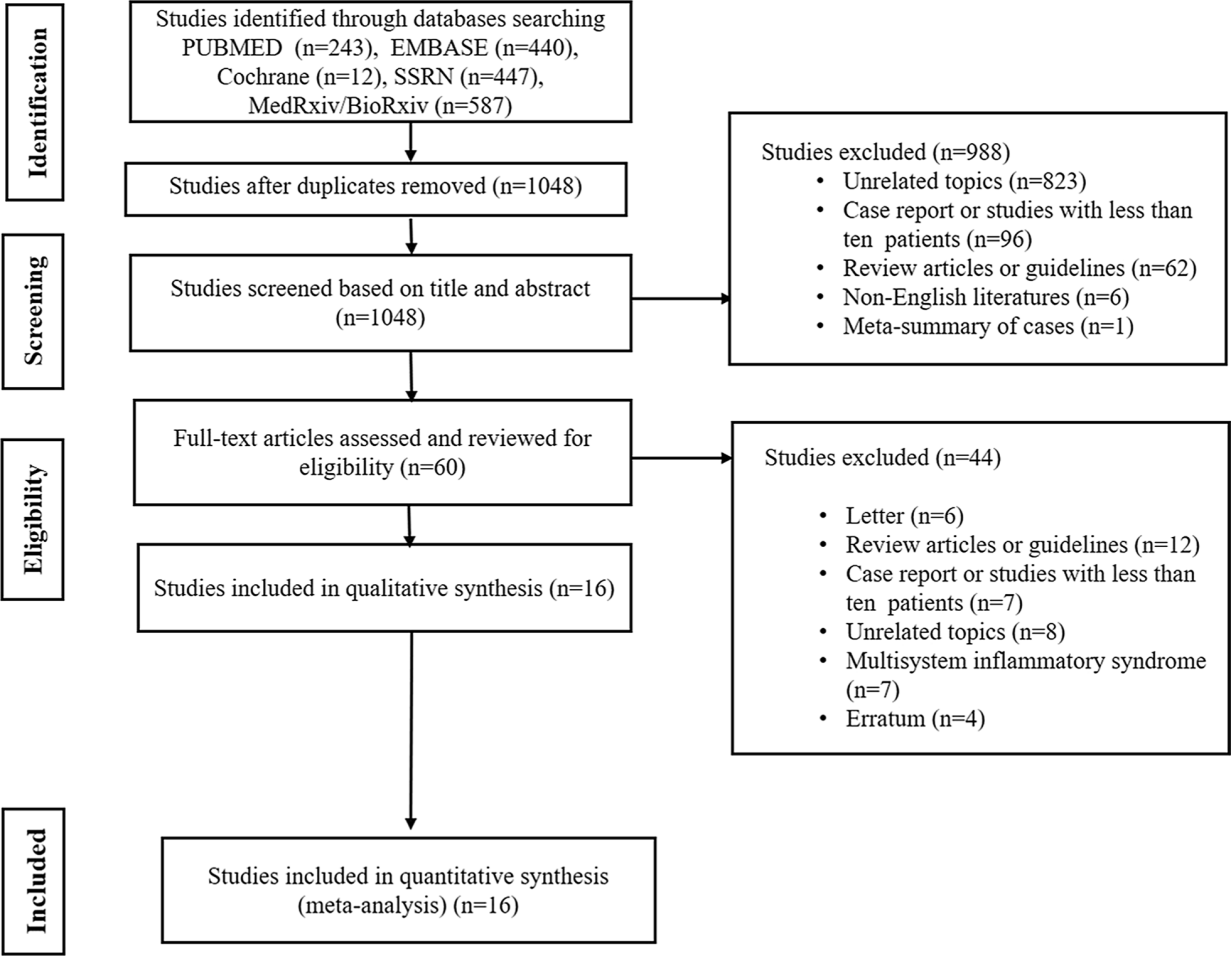
Flowchart of the literature review process

Two investigators independently reviewed the retrieved articles. A flowchart summarizing the literature search process is shown in Fig. 1. To determine the study eligibility, the full text of articles was evaluated for inclusion using the following criteria: (1) type of study, i.e., randomized controlled studies, prospective or retrospective cohort studies, and case–control studies with more than 10 patients; (2) study population, i.e., patients who recovered from COVID-19 and underwent CMR after recovery; and (3) primary outcome, i.e., the prevalence of abnormal CMR findings. Abnormal CMR findings included the presence of ventricular systolic dysfunction on cine imaging, the presence of myocardial or pericardial late gadolinium enhancement (LGE), abnormal signal intensity on T2-weighted (T2w) imaging, elevated native T1 or T2 values on the mapping sequence, a diagnosis of myocarditis based on the updated Lake Louise criteria, and the presence of pericardial effusion [9].

In contrast, a study was excluded if the study population was restricted to COVID-19 patients with multisystem inflammatory syndrome or reported CMR findings during the acute stage of COVID-19.

### Data extraction

Two investigators independently extracted data with disagreements resolved by consensus. The extracted parameters included the following: (a) article information and patient characteristics; (b) CMR protocol, i.e., CMR scanner type (1.5 or 3 T) and obtained CMR sequences including cine, parametric mapping (T1 and T2), LGE, and T2w; and (c) CMR findings, i.e., the number of patients with normal and abnormal CMR findings, abnormal cine findings (ventricular systolic dysfunction), elevated parametric mapping (native T1 and T2) and extracellular volume (ECV) values, presence of LGE (myocardial or pericardial), myocardial segments with abnormal T2 or LGE areas, myocardial LGE patterns (non-ischemic, ischemic, or dual) that fulfilled the diagnostic criteria for myocarditis on CMR based on the Lake Louise criteria [9], and presence of pericardial effusion. LGE at the right ventricular (RV) insertion points in the interventricular septum was not considered to indicate LGE presence because it is a common non-specific finding in athletes [27].

### Subgroup analysis

Subgroups were stratified according to (a) whether a patient group was limited to athletes and (b) levels of cardiac enzymes (troponin I or high-sensitivity troponin T) when CMR was performed. Studies wherein the cardiac enzyme data were not extractable were assigned to the “undetermined cardiac enzyme level” subgroup. An analysis of an “elevated cardiac enzyme level” subgroup could not be performed, because there were only seven patients in three studies who had elevated cardiac enzyme levels and extractable CMR findings [11, 28, 29].

### Quality assessment

Two investigators independently performed quality assessments of the selected studies using the Newcastle–Ottawa Quality Scale [30]: for each question within the Selection and Exposure/Outcome categories, the maximum score is 1, and for the Comparability category, the top score is 2. A study with a total score of 6 or higher was considered of “high quality.”

### Statistical analysis

The pooled prevalence and 95% confidence interval (CI) of each CMR finding were estimated using a generalized linear mixed model. The heterogeneity between studies was assessed using chi-square-based Q statistics and I^2^ statistics [31, 32], and significant heterogeneity was defined as a P-value of < 0.1 or an I^2^ value of > 50%. Subgroup analysis of the prevalence of CMR findings was performed for the “athlete” versus (vs.) “non-athlete” subgroups and the “normal cardiac enzyme level” vs. “undetermined cardiac enzyme level” subgroups. Meta-regression analysis was performed for major CMR parameters to investigate their contribution to a study’s heterogeneity, using the covariates “athlete” and “undetermined cardiac enzyme level.” Variables with P-values of < 0.2 in the univariable meta-regression analysis were included in the multivariable analysis. A P-value of < 0.05 was considered to indicate a statistically significant difference in the multivariable analysis. Publication biases were drawn as funnel plots and evaluated using the Egger test [33]. The analysis was performed using R (version 4.0.3; R Foundation for Statistical Computing, Vienna, Austria) with the “metafor” and “meta” packages [34, 35].

## Results

### Study characteristics

Following the literature search, 890 patients from 16 studies were included in this meta-analysis [11–14, 16–22, 28, 29, 36, 37]. Tables 1 and 2 summarize the study characteristics and CMR protocols of the included studies, respectively. A greater percentage of the included studies were conducted retrospectively (62.5%) at a single institution (93.8%). Most studies (81.3%) obtained cine, parametric mapping (native T1 and T2), and LGE sequences [11–14, 16–19, 21, 22, 28, 36, 37]. Similarly, nine studies obtained T2w sequences [11, 12, 16, 17, 20, 21, 28, 29, 36], and one study obtained a non-contrast-enhanced CMR without an LGE sequence [17].

**Table 1:** Study characteristics

First author (year)	Journal	Study design	Study sites (countries)	Patient description	Study period	Population (n)	Reason for exclusion (n)	Number of patients including in the analysis	Age (years)	Sex (n, male/female)	Diagnosis of COVID-19 by RT-PCR	Other tests for cardiac evaluation	Presence of cardiac symptoms at the time of CMR	Cardiac enzyme level at the time of CMR	Population restricted to athletes	CMR field strength	CMR scan time	CMR sequences
Ng et al. (2020)	*JACC Cardiovasc Imaging*	Retrospective, single-center, observational	Hong Kong	Recovered COVID-19 patients	NA	16	Ischemic etiology (1)	15	Median 68 (IQR: 53–69)	9/7	Yes	Troponin, CRP	Various (5/16)	Undetermined	No	1.5 T (GE)	Median 56 days after recovery	Cine, Mapping (T1 and T2), LGE
Huang et al. (2020)	*JACC Cardiovasc Imaging*	Retrospective, single-center, observational	China	Recovered COVID-19 patients	NA	26	None	26	Median 38 (IQR: 32–45)	10/16	Yes	hs-troponin I assay	Yes (26)	Normal (26)	No	3 T (Skyra, Siemens)	Median 47 days (IQR: 36–58) after symptom onset	Cine, T2WI, mapping (T1 and T2), LGE
Rajpal et al. (2020)	*JAMA Cardiol*	Prospective, single-center, observational	U.S	Athletes recovered from COVID-19	Between June 2020 and August 2020	26	None	26	Mean 19.5 (SD: 1.5)	16/10	Yes	ECG, troponin I assay, echocardiography	Various (12/26)	Normal (26)	Yes	1.5 T (Magnetom Sola, Siemens)	11–53 days after recommended quarantine	Cine, mapping (T1 and T2), LGE, ECV
Knight et al. (2020)	*Circulation*	Retrospective, single-center, observational	England	Recovered COVID-19	Until April 2020	51	Acute coronary syndromes (6) pulmonary emboli (12), or known cardiac pathology (7)	29	Mean (SD) 64 (9)	24/5	Yes	NR	Yes (29)	Undetermined	No	1.5 T (Avanto Aera;Siemens)	Mean 46 days after symptom onset	Cine, Mapping (T1 and T2), LGE, Adenosine stress perfusion
Puntmann et al. (2020)	*JAMA Cardiol*	Prospective, single-center, observational	Germany	Recovered COVID-19 patients	Between April 2020 and June 2020	100	None	100	Mean 49 (SD: 14)	53/47	Yes	Hs-troponin T assay	Various (36/100)	Undetermined	No	3 T (Skyra, Siemens)	Median 71 (IQR 64–92) after COVID-19 diagnosis	Cine, Mapping (T1 and T2) LGE
Eiros et al. (2020)	*MedRxiv*	Retrospective, single-center observational	Spain	Recovered COVID-19 patients (health care workers)	Between May 25, 2020 and June 12, 2020	142	Claustrophobia (1),history of hypertrophic myocardiopathy (1), inherited immune deficiency (1)	139	Median 52 (IQR: 41–57)	39/100	103 diagnosed by RT-PCR, 36 by serology	ECG; NT-pro-BNP and hs-troponin T assays	Various (91/139)	Normal (138), elevated (1)	No	1.5 T (Achiva, Philips)	Median 10.4 (IQR: 9.3–11.0) weeks after symptom onset^b^	Cine, T2WI Mapping (T1 and T2), LGE
Vago et al. (2020)	*JACC Cardiovasc Imaging*	Retrospective, single-center observational	Hungary	Athletes recovered from COVID-19	NA	12	None	12	Median 23 (IQR: 20–23)	2/10	Yes	CRP, NT-pro-BNP, and hs-troponin T assays	Yes (12)	Normal (11), undetermined (1)	Yes	1.5 T (Magnetom, Aera, Siemens)	Median 17 (IQR: 17–19) days after positive PCR in 10 female athletes, 67 and 90 days in 2 male athletes	Cine, T2WI, mapping (T1 and T2), LGE
Brito et al. (2020)	*JACC Cardiovasc Imaging*	Retrospective, single-center observational	U.S	Student athletes recovered from COVID-19	By August 2020	54	Claustrophobia (1), no CMR (5)	48	Median 19 (range 19–21)^a^	46/8^a^	PCR or antibody test	Echocardiography, troponin I assay, ECG	Various (37/48)	Undetermined	Yes	1.5 T (Magnetom, Aera; Siemens)	Median 27 days (range 22–33 days) from diagnosis of COVID-19	Cine, T2WI, mapping (T1 and T2), LGE
Clark et al. (2021)	*Circulation*	Retrospective, single-center, observational	U.S	Athletes recovered from COVID-19	Since August 2020	22	None	22	Median 20	9/11	Yes	ECG, troponin I assay, echocardiography	NR	Normal (18)	Yes	1.5 T (Avanto fit, Siemens)	Median 52 days after COVID-19 diagnosis	Cine, mapping (T1 and T2), LGE, ECV
Malek et al. (2021)	*J Magn Reson Imaging*	Retrospective, single-center observational	Germany	Student athletes recovered from COVID-19	Diagnosed COVID-19 between August and October 2020	26	None	26	Median 19 (IQR 19–21)	5/21	Yes	ECG, CRP, hs-troponin I assay	NR	Normal (26)	Yes	1.5 T (Magnetom Avanto Fit, Siemens)	Median 32 days (IQR 22–62 days) after diagnosis	Cine, T2WI, Mapping (T1 and T2), LGE
Li et al. (2021)	*Radiology*	Prospective, single-center, observational	China	Recovered COVID-19 patients	Between May and September 2020	78	Due to discharge < 90 days (n = 5), abnormal cardiac enzyme (n = 3), abnormal ECG findings (n = 4), not underwent CMR (n = 16), history of cardiovascular disease or HTN (7), contrast allergy (1), image quality (2)	40	Mean 54 (SD: 12)	24/160	Yes	ECG, CRP, CK, CKMB Troponin I assays	No	Normal (40)	No	3 T (Skyra, Siemens)	Mean 124 ± 17 days after discharge, Mean 158 ± 18 after admission	Cine, LGE, Strain
Starekova et al. (2021)	*JAMA Cardiology*	Retrospective, single-center observational	U.S	Athletes recovered from COVID-19	Between January 1, 2020, and November 29, 2020	145	none	145	Mean 20 (range: 17–23)	108/37	Yes	ECG, Troponin I, NT-proBNP, CRP, ESR assays and echocardiography	Various (1/145)	Normal (141), elevated (2)	Yes	1.5 T or 3 T (GE)	Median 15 days after diagnosis	Cine, T2WI, Mapping (T1 and T2), LGE
Wang et al. (2021)	*J Cardiovasc Magn Reson*	Prospective, single-center, observational	China	Recovered COVID-19 patients	From May 8 to July 20, 2020	47	History of cardiovascular disease (3)	44	Mean 47.6 (SD: 13.3)	19/25	Yes	NR	NR	Undetermined	No	3 T (Ingenia, Philips)	Mean 102.5 ± 20.6 days after diagnosis	Cine, T2WI, T2 star map, LGE, strain
Pan et al. (2021)	*J Magn Reson Imaging*	Prospective, single-center observational	China	Recovered COVID-19 patients	Between March 2020 and April 2020	31	History of cardiovascular disease, presence of cardiac symptoms, or elevated cardiac enzymes (10)	21	Median 36 (IQR: 31–47)	10/11	Yes	NR	No	Undetermined	No	3 T (Signa, GE)	Median 46 day (IQR 43–50 days)	Cine, T2WI, Mapping (T1 and T2)
Zhou et al. (2021)	*Plos one*	Prospective, single-center, observational	Hong Kong	Recovered COVID-19 patients	Diagnosed up to April 2020	97	No CMR (85)	12	Mean 46.5 (SD:18.6)^a^	52/45^a^	Yes	ECG, Troponin I, NT-proBNP assay and echocardiography	NR	Normal (7), elevated (4)	No	NR	NR	Cine, T2WI, LGE
Kotecha et al. (2021)	*Eur Heart J*	Retrospective, Multicenter study	U.K	Recovered COVID-19 patients	Discharged up to 20 June 2020	820	No CMR (672)	148	Mean 64 (SD:12)	104/44	Yes	NR	NR	Undetermined	No	1.5 T (Magnetom, Aera, Siemens)	Median 56 days (IQR 30–88 days) after discharge	Cine, Mapping (T1 and T2), LGE, stress perfusion

*CMR* cardiovascular magnetic resonance imaging, *CRP* C-reactive protein, *ECG* electrocardiography, *ECV* extracellular volume, *hs-troponin T* high-sensitivity troponin T, *IQR* interquartile range, *LGE* late gadolinium enhancement, *NA* not available, *NR* not reported, *NT-pro-BNP* N-terminal pro-natriuretic peptide, PCR polymerase chain reaction, RT-PCR real-time polymerase chain reaction, *SD* standard deviation, *T2w* T2-weighted imaging, *US* United States, *WBC* white blood count

^a^Only provided value of the entire study population

^b^Median 9.4 weeks (IQR: 8.1–10.0 weeks) and median 4.4 weeks (IQR: 3.6–5.0 weeks) after the positive RT-PCR test and diagnosed through antibodies testing, respectively

**Table 2:** Cardiovascular magnetic resonance findings of the included studies

First author (year)	CMR abnormality, n (%)	Fulfilled diagnostic criteria of myocarditis on CMR^a^ (n)	Cine abnormality (n)	T1 mapping abnormality (n)	T2w abnormality (n)	T2 mapping abnormality (n)	T2 segment	T2 abnormality (T2w or T2 map)	ECV abnormality (n)	Myocardial LGE (n)	Pericardial LGE (n)	Total LGE	LGE segment	LGE pattern	Increased T2 value without LGE (n)	LGE without T2 elevation (n)	Pericardial effusion (n)
Ng et al. (2020)	9 (66.7%)	4	NR	5	NA	5	Global	5	NR	3	NR	3	NR	Non-ischemic (3)^a^	2	1	0
Huang et al. (2020)	15 (57.7%)	7	NR	NR	14	NR	NR	14	NR	8	0	8	Inferior or lateral at the mid and basal segments	Focal linear subepicardial and patchy mesocardial	7	1	7
Rajpal et al. (2020)	13 (50%)	4	1	0	NA	4	Mid-inferoseptal (3) mid-anteroseptal (2), basal inferoseptal (1)	4	1	12	0	12	Septal (19), inferior or lateral (5) at the mid and basal segments	Patchy (6), linear (3), epicardial (1), RV insertion (2)	0	8	2
Knight et al. (2020)	20 (69%)	NR	2	NR	NA	0	NR	0	NA	20	0	20	NR	Non-ischemic (11), ischemic (5), dual (4)	NR	NR	2
Puntmann et al. (2020)	78 (78%)	NR	NR	73	NA	60	NR	60	NR	32	22	NR	NR	Nonischemic (20), ischemic (12), pericardial (22)	NR	NR	20
Eiros et al. (2020)	104 (75%)	51	7	58	6	6	NR	NR	52	10	0	10	NR	NR	NR	NR	42
Vago et al. (2020)	0 (0%)	0	0	0	0	0	NR	0	NA	0	0	0	NR	NR	0	0	NR
Brito et al. (2020)	26 (54.2%)	0	1	9	0	0	NR	0	NA	1	19	NR	Lateral	Pericardial (19), myocardial (1)	0	1	28
Clark et al. (2021)	4 (6.8%)	2	0	NA	NA	1	Mid septum	NR	NA	3	1	4	NR	NR	NA	NA	NA
Malek et al. (2021)	7 (26.9%)	0	2	0	3	1	NR	4	0	1	0	1	Inferolateral segment	Mid wall	4	1	2
Li et al. (2021)	24 (60%)	NR	NA	NA	NR	NA	NR	NA	24	1	0	1	Mid-inferior segment	NR	NR	NR	NA
Starekova et al. (2021)	4 (2.8%)	2	NA	2/141	2	1/102	Apical inferolateral, and basal inferior segment	2	NR	4	1	4	Apical inferolateral, and basal inferior segment	Mid myocardial and subepicardial (1), epicardial (1), mid myocardial (2)	0	2	1
Wang et al. (2021)	13 (29.5%)	NA	NA	NA	NA	NA	NA	NA	NR	13	0	13	Inferior wall and inferior-lateral wall of the basal segment	Mid myocardium, subepicardium	NA	NA	NA
Pan (2021)	15 (71.4%)	NA	3	5	NR	10	NR	10	NR	NR	NR	NR	NR	NR	NR	NR	NA
Zhou (2021)	1 (8.3%)	0	0	NR	0	NR	NR	0	NR	1	0	1	Basal anterolateral segment	Subepicardial	0	1	NA
Kotecha (2021)	80 (54.1%)	12	17	23/137	NR	12/137	NR	12/137	NR	70/144	0	70/144	NR	Subepicardial (28), midwall (14), subendocardia and subepicardial (3), subendocardial and midwall (2)	NA	NA	8

*ECV* extracellular volume, *LGE* late gadolinium enhancement, *NA* not available, *NR* not reported, *RV* right ventricular, *T2w* T2-weighted imaging

^a^One patient who showed ischemic LGE with a history of myocardial infarction was excluded

Six of the 16 included studies enrolled only athletes as participants [16, 19, 21, 28, 36, 37], whereas there was no restriction on the occupation of study participants in the other 10 studies [11–18, 20, 29]. Eight studies had populations with normal cardiac enzyme levels [11, 12, 15, 16, 19, 28, 29, 37]. Seven other studies had patients with undetermined cardiac enzyme levels [13, 14, 17, 18, 20–22], and one study reported data for normal and undetermined cardiac enzyme level subgroups [36].

### Pooled prevalence of abnormal CMR findings

**Fig. 2:**
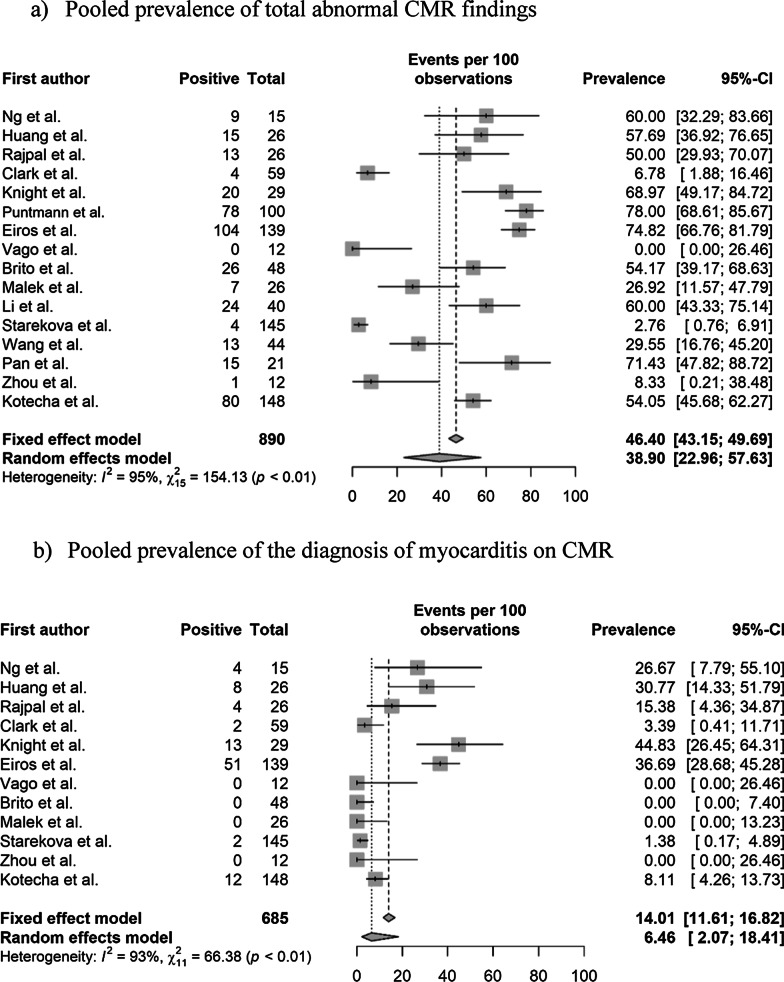

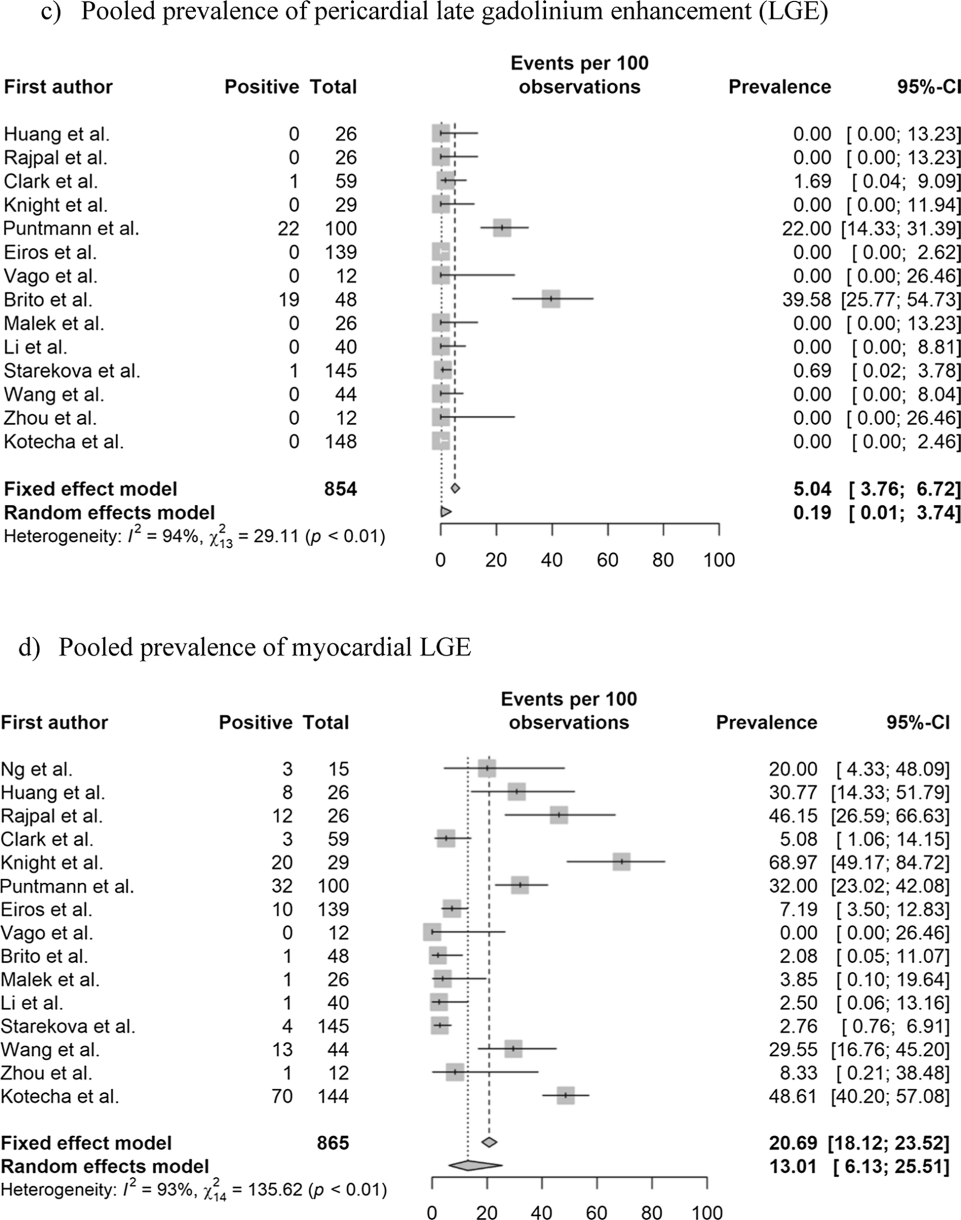

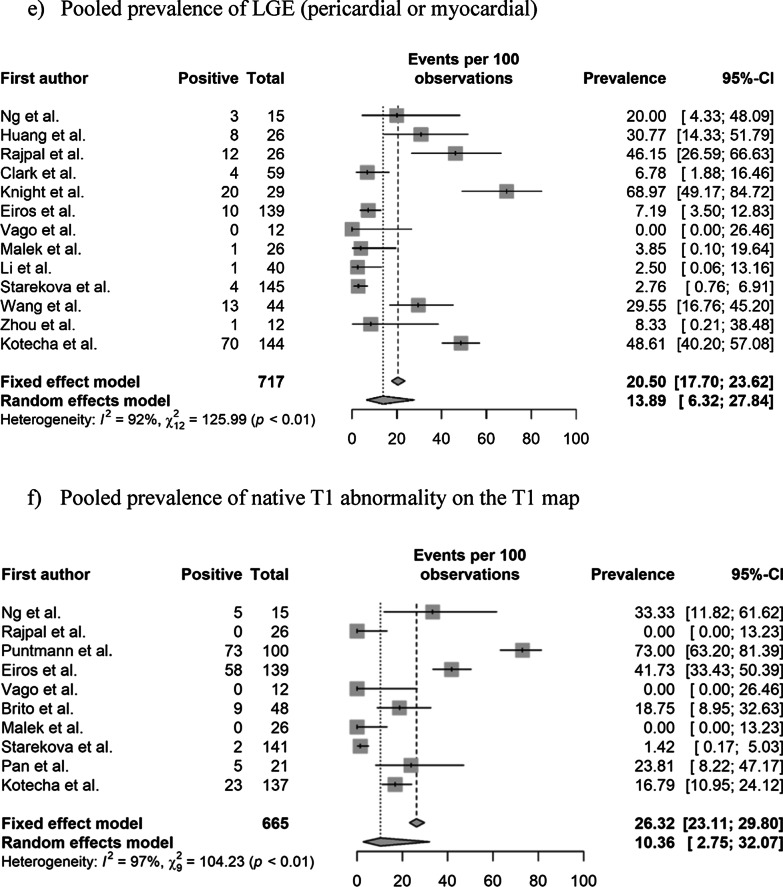

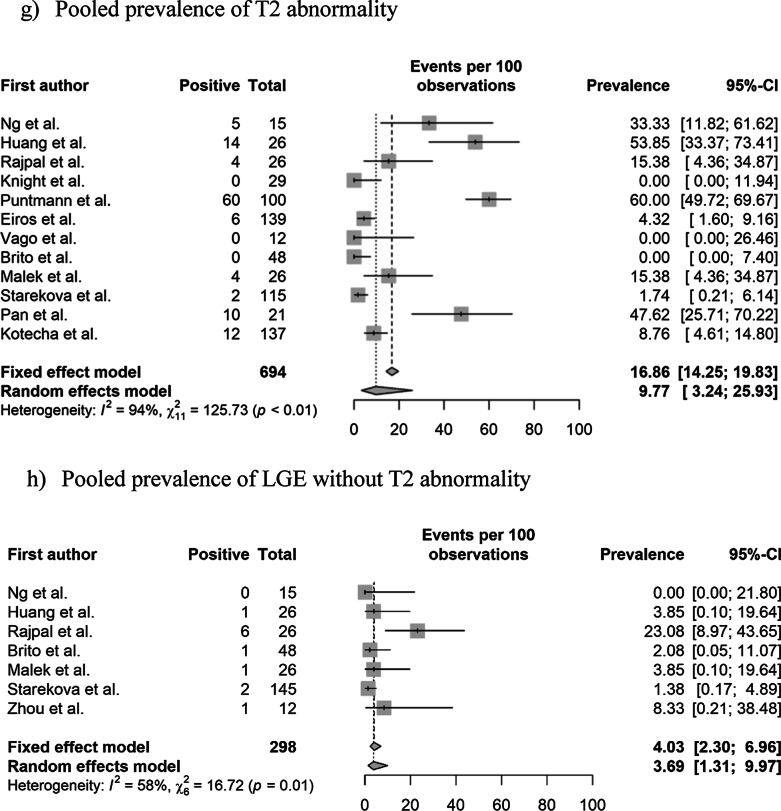

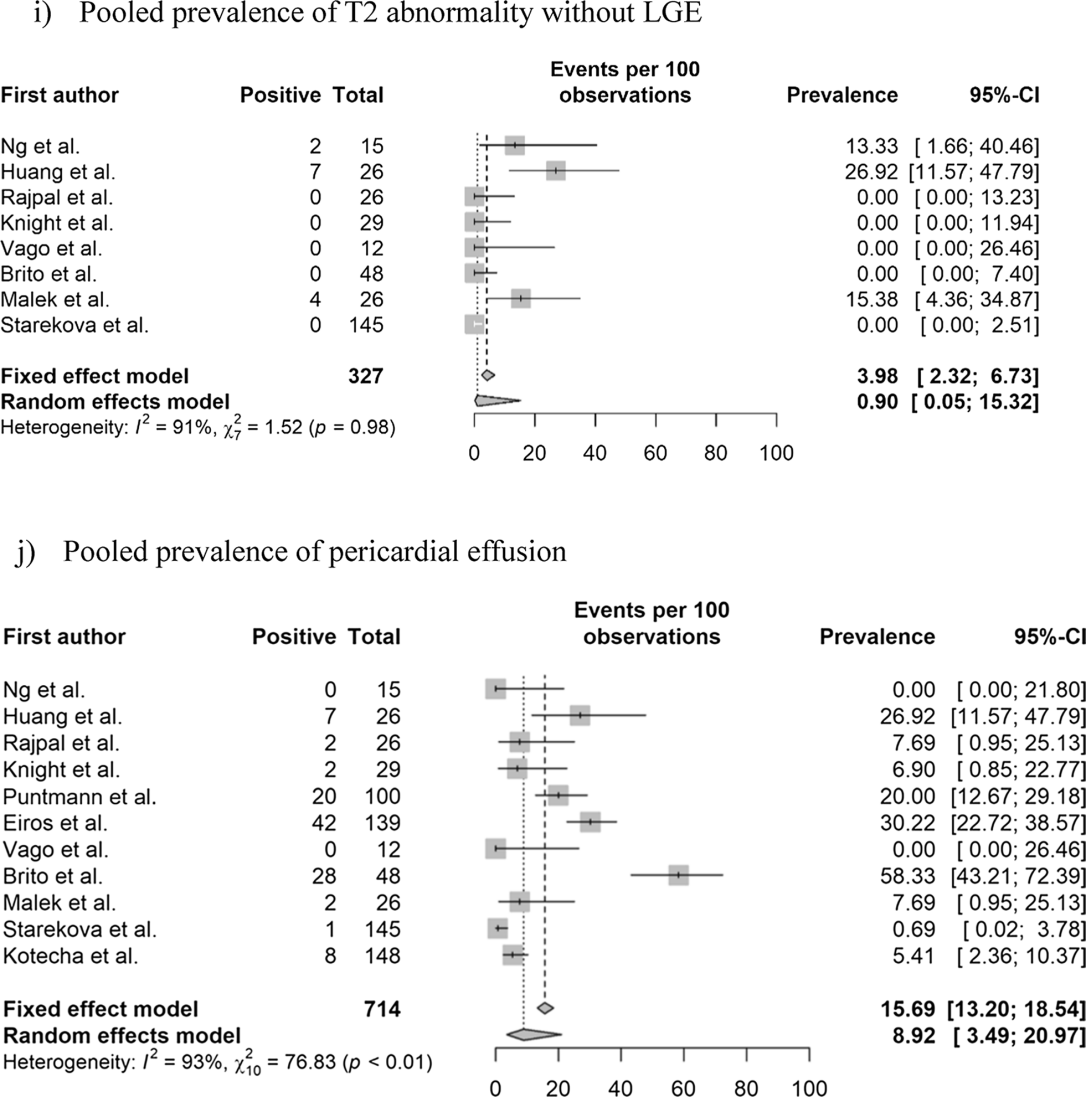

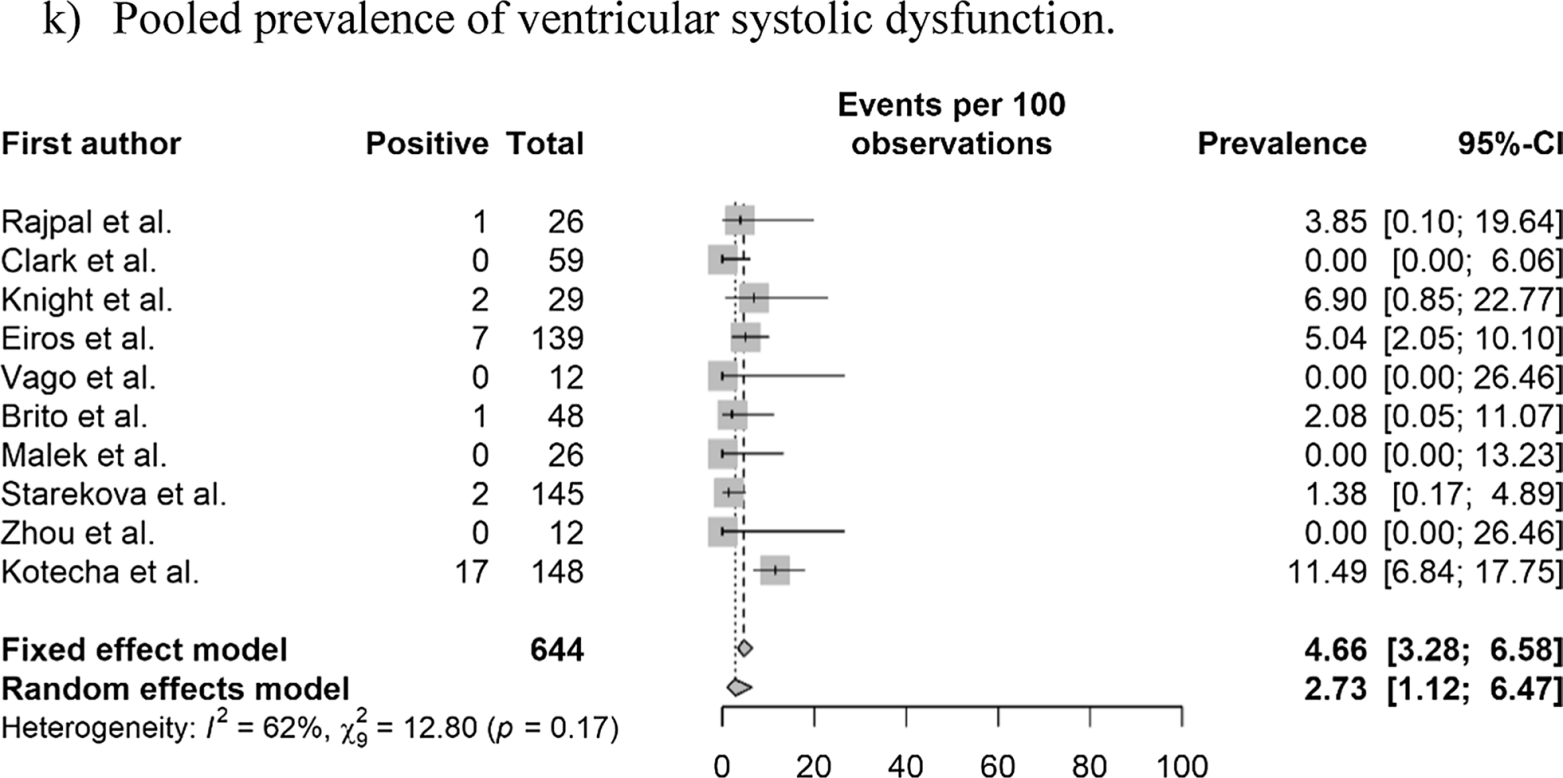
Pooled prevalence of abnormal CMR findings in patients who recovered from COVID-19. **a** Pooled prevalence of total abnormal CMR findings. **b** Pooled prevalence of the diagnosis of myocarditis on CMR. **c** Pooled prevalence of pericardial late gadolinium enhancement (LGE). **d** Pooled prevalence of myocardial LGE. **e** Pooled prevalence of LGE (pericardial or myocardial). **f** Pooled prevalence of native T1 abnormality on the T1 map. **g** Pooled prevalence of T2 abnormality. **h** Pooled prevalence of LGE without T2 abnormality. **i** Pooled prevalence of T2 abnormality without LGE. **j** Pooled prevalence of pericardial effusion. **k** Pooled prevalence of ventricular systolic dysfunction. CMR, cardiovascular magnetic resonance; COVID-19, coronavirus disease 2019; LGE, late gadolinium enhancement

The pooled prevalence values of abnormal CMR findings are summarized in Table 3 and Fig. 2. The overall prevalence of any abnormal CMR finding in recovered COVID-19 patients was 46.4% (95% CI 43.2%–49.7%) in 16 studies [11–22, 28, 29, 36, 37]. The pooled prevalence of a CMR diagnosis of myocarditis was 14.0% (95% CI 11.6%–16.8%) in 12 studies [11–14, 16, 19, 21, 22, 28, 29, 36, 37]. The pooled prevalence of pericardial and myocardial LGE was 5.0% (95% CI 3.8%–6.7%) in 14 studies [11–16, 18–21, 28, 29, 36, 37] and 20.7% (95% CI 18.1%–23.5%) in 15 studies [11–16, 18–22, 28, 29, 36, 37], respectively. The pooled prevalence of total (pericardial or myocardial) LGE was 20.5% (95% CI 17.7%–23.6%) in 13 studies [11–16, 19, 20, 22, 28, 29, 36, 37].

**Table 3:** Pooled prevalence of abnormal CMR findings

Parameter (number of studies)	Overall		Study population				Cardiac enzyme level^†^			
	Prevalence (%)	Heterogeneity^*^	Non-athletes (n = 10)		Athletes (n = 6)		Normal (n = 9)		Undetermined (n = 8)	
			Prevalence (%)	Heterogeneity^*^	Prevalence (%)	Heterogeneity^*^	Prevalence (%)	Heterogeneity^*^	Prevalence (%)	Heterogeneity^*^
Abnormal CMR findings (n = 16)	46.4 [43.2–49.7]	< 0.01, 95%	62.5 [58.5–66.4] (n = 10)	< 0.01 86%	17.1 [13.3–21.7] (n = 6)	< 0.01, 92%	35.9 [31.7–40.3] (n = 9)	< 0.01, 95%	59.4 [54.5–64.04] (n = 8)	< 0.01, 77%
Diagnosis of myocarditis on CMR (n = 12)	14.0 [11.6–16.8]	< 0.01, 93%	23.9 [19.8–28.5] (n = 6)	< 0.01, 85%	2.5 [01.3–5.0] (n = 6)	0.11, 64%	15.2 [12.1–19.0] (n = 8)	< 0.01, 90%	12.0 [8.5–16.8] (n = 5)	< 0.01, 90%
Presence of pericardial LGE (n = 14)	5.0 [3.8–6.7]	< 0.01, 94%	4.1 [2.7–6.1] (n = 8)	> 0.99, 98%	6.7 [4.4–10.0] (n = 6)	< 0.01, 86%	10.4 [7.1–15.1] (n = 5)	< 0.01, 88%	24.8 [20.6–29.6] (n = 7)	< 0.01, 96%
Presence of myocardial LGE (n = 15)	20.7 [18.1–23.5]	< 0.01, 93%	28.8 [25.2–32.7] (n = 9)	< 0.01, 92%	6.7 [4.4–10.0] (n = 6)	< 0.01, 82%	8.6 [6.0–12.1] (n = 8)	< 0.01, 83%	36.5 [31.8–41.4] (n = 7)	< 0.01, 93%
Presence of LGE (myocardial or pericardial) (n = 13)	20.5 [17.7–23.6]	< 0.01, 92%	28.1 [24.1–32.4] (n = 8)	< 0.01, 92%	7.8 [5.2–11.7] (n = 5)	< 0.01, 84%	8.3 [5.8–11.8] (n = 8)	< 0.01, 85%	45.5 [39.2–51.9] (n = 5)	< 0.01, 75%
Increased native T1 value on the T1 map (n = 10)	26.3 [23.1–29.8]	< 0.01, 97%	39.8 [35.2–44.6] (n = 5)	< 0.01, 92%	4.4 [2.4–7.7] (n = 5)	0.02, 81%	1.0 [0.1–8.7] (n = 4)	> 0.99, 0%	35.7 [30.7–41.1] (n = 6)	< 0.01, 88%
T2 abnormality (n = 12)	16.9 [14.3–19.8]	< 0.01, 94%	22.9 [19.3–26.9] (n = 7)	< 0.01, 95%	4.4 [2.4–8.0] (n = 5)	0.08, 75%	10.4 [7.1–15.1] (n = 5)	< 0.01, 88%	24.8 [20.6–29.6] (n = 7)	< 0.01, 96%
T2 abnormality without LGE (n = 8)	4.0 [2.3–6.7]	0.98, 91%	12.9 [6.8–22.9] (n = 3)	0.061, 73%	1.6 [0.6–4.1] (n = 5)	> 0.99, 90%	5.7 [3.3–9.5] (n = 5)	< 0.01, 83%	2.2 [0.5–8.2] (n = 4)	> 0.99, 76%
Presence of myocardial LGE without T2 abnormality (n = 7)	4.0 [2.3–7.0]	0.01, 58%	3.8 [0.2–46.6] (n = 3)	0.85, 0%	4.1 [2.2–7.4] (n = 4)	< 0.01, 71%	4.4 [2.4–8.0] (n = 5)	< 0.01, 64%	1.6 [0–100] (n = 2)	> 0.99, 0%
Pericardial effusion (n = 11)	15.7 [13.2–18.5]	< 0.01, 93%	17.3 [14.1–21.0] (n = 6)	< 0.01, 86%	12.8 [9.3–17.5] (n = 5)	< 0.01, 89%	5.2 [3.0–9.0] (n = 5)	< 0.01, 75%	17.0 [13.4–21.4] (n = 6)	< 0.01, 91%
Ventricular systolic dysfunction on cine (n = 10)	4.7 [3.3–6.6]	0.17, 62%	7.4 [2.9–17.3] (n = 4)	0.28, 27%	1.3 [0.4.–4.5] (n = 6)	0.98, 0%	NA	NA	7.4 [3.1–16.8] (n = 4)	0.34, 22%

Numbers in brackets represent 95% confidence intervals

*CMR* cardiovascular magnetic resonance, *LGE* late gadolinium enhancement, *n* number of studies

^*^Values indicate p-values for the Cochran Q test and I^2^

The pooled prevalence of an elevated native T1 was 26.3% (95% CI 23.1%–29.8%) in 10 studies [11, 14, 16–19, 21, 22, 28, 36] and that of a T2 abnormality (increased T2 value on the T2 map or abnormal SI on T2 weighted (T2w) imaging was 16.9% (95% CI 14.3%–19.8%) in 12 studies [11–14, 16–19, 21, 22, 28, 36]. The pooled prevalence of a T2 abnormality without LGE was 4.0% (95% CI 2.3%–6.7%) in eight studies [12, 13, 16, 19, 21, 22, 28, 36], and that of LGE without a T2 abnormality was 4.0% (95% CI 2.3%–7.0%) in seven studies [12, 16, 19, 21, 22, 28, 29]. The pooled prevalence of pericardial effusion was 15.7% (95% CI 13.2%–18.5%) in 11 studies [11–14, 16, 18, 19, 21, 22, 28, 36], and that of ventricular systolic dysfunction on cine CMR was 4.7% (95% CI 3.3%–6.6%) in 10 studies [11, 13, 14, 16, 19, 21, 28, 29, 36, 37]. Significant heterogeneities among the included studies were observed for all parameters of abnormal findings (I^2^ > 50%).

### Prevalence of abnormal CMR findings relative to patient characteristics

The pooled prevalence values of abnormal CMR findings within subgroups are summarized in Table 3.

#### Non-athletes vs. athletes

Of the 890 patients in 16 studies, 316 (35.5%) subjects were athletes [16, 19, 21, 28, 36, 37]. The pooled prevalence of abnormal CMR findings and a CMR diagnosis of myocarditis was higher in non-athletes than in athletes (62.5% vs. 17.1% and 23.9% vs. 2.5%, respectively). Similarly, compared with athletes, non-athletes had a higher pooled prevalence of other CMR abnormalities, including myocardial LGE (28.8% vs. 6.7%), an elevated native T1 (39.8% vs. 4.4%), a T2 abnormality (22.9% vs. 4.4%), a T2 abnormality without LGE (12.9% vs. 1.6%), pericardial effusion (17.3% vs. 12.8%), and ventricular systolic dysfunction (7.4% vs. 1.3%). In contrast, the pooled prevalence values were slightly higher in athletes than in non-athletes for pericardial LGE (6.7% vs. 4.1%) and were similar in both groups for myocardial LGE without T2 abnormality (4.1% vs. 3.8%). After subgroup analysis, the heterogeneity of studies became insignificant for abnormal CMR and ventricular dysfunction in both subgroups and the presence of myocardial LGE without T2 abnormality in the non-athlete subgroup (all, p > 0.1, I^2^ < 50%).

#### Normal cardiac enzyme level vs. undetermined cardiac enzyme level

Among the 890 patients in 16 studies, 474 (53.3%) from nine studies [11, 12, 15, 16, 19, 28, 29, 36, 37] had normal enzyme levels (e.g. troponin) and 406 (45.6%) from eight studies had undetermined cardiac enzyme levels [13, 14, 17, 18, 20–22, 36]. The undetermined cardiac enzyme level subgroup exhibited a higher pooled prevalence than the normal cardiac enzyme level subgroup for abnormal CMR findings (59.4% vs. 35.9%), the presence of pericardial (24.8% vs. 10.4%) or myocardial LGE (36.5% vs. 8.6%), an elevated native T1 value (35.7% vs. 1%), T2 abnormality (24.8% vs. 10.4%), and pericardial effusion (17% vs. 5.2%). In contrast, the pooled prevalence values were higher in the normal cardiac enzyme level subgroup than in the undetermined cardiac enzyme level subgroup for a diagnosis of myocarditis on CMR (15.2% vs. 12.0%) and the presence of myocardial LGE without T2 abnormality (4.4% vs. 1.6%). After subgroup analysis, the heterogeneity between studies became insignificant for ventricular dysfunction in the undetermined enzyme level subgroup (p = 0.34, I^2^ = 22%).

Meta-regression analysis results are summarized in Table 4. In the univariable meta-regression analyses, the athlete subgroup was significantly associated with heterogeneity for abnormal CMR findings, myocarditis diagnosis on CMR, myocardial LGE, and a T2 abnormality (all, p < 0.2). In contrast, undetermined cardiac enzyme level was significantly associated with heterogeneity for abnormal CMR findings and the presence of myocardial LGE (all, p < 0.2). In the multivariable meta-regression analyses, being an athlete was a significant independent factor associated with heterogeneity for abnormal CMR findings (p < 0.05). However, undetermined cardiac enzyme levels were not significantly associated with heterogeneity in multivariable meta-regression analyses.

**Table 4:** Meta-regression analysis for prevalence of each CMR finding

Parameter	Univariable meta-regression analysis		Multivariable meta-regression analysis	Residual heterogeneity after multivariable meta-regression analysis
	p-value	I^2^	p-value	I^2^
Abnormal CMR findings				92.8%
Athlete group	0.002	93.4%	0.018	
Undetermined cardiac enzyme level group	0.061	94.6%	0.173	
Diagnosis of myocarditis on CMR				N/A
Athlete group	< 0.001	90.6%	N/A	
Undetermined cardiac enzyme level group	0.405	93.7%	N/A	
Presence of myocardial LGE				93.9%
Athlete group	0.050	95.4%	0.206	
Undetermined cardiac enzyme level group	0.033	94.0%	0.138	
T2 abnormality				N/A
Athlete group	0.035	97.2%	N/A	
Undetermined cardiac enzyme level group	0.629	96.9%	N/A	
Pericardial effusion				N/A
Athlete group	0.753	93.8%	N/A	
Undetermined cardiac enzyme level group	0.353	92.1%	N/A	

*CMR* cardiovascular magnetic resonance, *NA* not available

### Quality of the studies

The quality assessments of the included studies are summarized in Additional file 1: Table S1. Most studies were classified as “high quality” (87.5% of the studies received scores of 6 or 7, and 12.5% received a score of 5).

### Systematic review of the ECV, patterns of LGE, and cine findings

#### ECV findings

Six studies reported that ECV was significantly higher in recovered COVID-19 patients than in healthy controls [11, 12, 15, 16, 19, 37]. Huang et al. showed that ECV was significantly higher in recovered COVID-19 patients who showed abnormal CMR findings than in controls (median ECV: 28.2% vs. 23.7%, p = 0.001) [12]. Eiros et al. reported that the prevalence of elevated ECV was 37.4% (52/139) in recovered COVID-19 patients [11]. Li et al. reported that ECV was significantly elevated in patients recovered from moderate (median ECV, 29.7%) or severe COVID-19 (median ECV, 31.4%) relative to healthy controls (median ECV 25%, p < 0.001) and that the prevalence of elevated ECV was 60% (24/60) in recovered COVID-19 patients [15]. Three studies on athlete participants reported a relatively lower prevalence of abnormal ECV (Rajpal et al.: 3.8%, 1/26; Clark et al.: 4.5%, 1/22; Malek et al.: 0%, 0/26) than two studies on non-athletes (Eiros et al.: 37.4%, Li et al.: 60%) [16, 19, 37].

#### Patterns of LGE, the involved segments of LGE, and T2 abnormalities on CMR

A non-ischemic LGE pattern was the most frequent pattern of myocardial LGE reported in 11 studies (87.9%, 123/140, Table 2) [12–14, 16, 18–20, 22, 28, 29, 37]. Specifically, subepicardial, epicardial, and mid-wall LGE were the patterns reported in these studies. Frequently reported myocardial LGE locations in eight studies included the mid and basal inferior, septal, and lateral segments [14–17, 19, 20, 29, 37].

Two studies reported eight locations of T2 abnormalities in six patients [19, 28]. Similar to the LGE location, the mid-inferoseptum (37.5%, 3/8) and mid-anteroseptum (25%, 2/8) were the most common locations reported in the study by Rajpal et al. [19]. A study by Clark et al. on athletes reported that the T2 value was significantly higher in athletes who recovered from COVID-19 than healthy athlete controls (p = 0.02) [37].

#### Ventricular systolic dysfunction on cine sequence

Among the 16 included studies, six were excluded from the meta-analysis for ventricular dysfunction because the prevalence could not be extracted [12, 13, 15, 18, 20, 28]. Four studies reported that significant RV dysfunction was observed in recovered COVID-19 patients [12, 17, 19, 37]. Huang et al. reported that the RV ejection fraction (RVEF) was significantly lower in recovered COVID-19 patients with abnormal CMR findings than in healthy controls (RVEF 36.5% vs. 46.1%, p = 0.01). In contrast, the left ventricular (LV) ejection fraction (LVEF) was low in only one patient (3.9%, 1/26) with abnormal CMR findings [12]. Pan et al. reported a decrease in RVEF in two patients (9.5%), and the mean RVEF was significantly lower in recovered COVID-19 patients than in controls (p < 0.05). However, the mean LVEF was similar between recovered COVID-19 patients and controls [17].

LV or biventricular dysfunction in recovered COVID-19 patients has been evaluated in previous studies [11, 13, 18, 21]. Puntmann et al. measured and reported that the LVEF and RVEF were significantly lower in recovered COVID-19 patients than in matched controls (LVEF: 57% vs. 62%; RVEF: 54% vs. 59%) (all, p < 0.05) [18]. Malek et al. and Eiros et al. reported that the prevalence of LV systolic dysfunction in recovered COVID-19 patients was 8% and 5%, respectively.

Malek et al. reported that two athletes (8%) exhibited an enlarged LV with a slightly decreased LVEF, whereas RVEF was normal [16]. Although Eiros et al. reported LV wall motion abnormalities in seven patients (5%, 7/139), data on RV function were not provided [11]. Although ventricular systolic function was normal, abnormal strain values were reported in two studies [15, 20]. Li et al. reported that global LV longitudinal strain was significantly lower in patients who recovered from moderate or severe COVID-19 than in healthy controls (moderate COVID-19 group: − 12.5%; severe COVID-19 group: − 12.5%; healthy controls: − 15.4%; p = 0.002 and p = 0.001, respectively) [15]. Wang et al. reported that recovered COVID-19 patients with LGE had significantly lower peak global circumferential strain values in the LV and RV and lower peak global longitudinal strain values in the RV than recovered COVID-19 patients with no LGE or healthy controls (both, p < 0.05) [20]. No cine abnormalities were reported in the populations studied by Vago et al., Ng et al. and Kotecha et al. [13, 14, 36].

### Publication bias

Funnel plots of the prevalence values of abnormal CMR findings, a diagnosis of myocarditis on CMR, myocardial LGE, a T2 abnormality, and pericardial effusion are presented in Additional file 1: Fig. S1. All parameters had symmetric funnel plots without significant publication bias (p > 0.05), except for T2 abnormality without LGE (p = 0.04).

## Discussion

This meta-analysis revealed that nearly half of the patients exhibited one or more abnormal CMR findings after recovery from COVID-19. Athletes and patients in the normal cardiac enzyme level subgroups showed a lower prevalence of abnormal CMR findings than non-athletes and patients in the undetermined cardiac enzyme level subgroups. The most frequent abnormal CMR finding was the presence of an elevated native T1 value on the T1 map (26.3%), followed by a presence of myocardial LGE (20.7%).

Non-invasive CMR is a valuable diagnostic tool to evaluate the presence and extent of myocardial injury in COVID-19 patients [9]. A previously published systematic review reported CMR findings for 199 COVID-19 patients, including patients with myocarditis (40.2%), myopericarditis, stress-induced cardiomyopathy, and ischemia [10]. However, the studies included in this systematic review primarily conducted CMR during the active phase of COVID-19 [10]. Therefore, the data did not contribute to our understanding of whether myocardial inflammation or scarring would be observed on CMR in recovered COVID-19 patients.

Patients with myocarditis may develop arrhythmia or heart failure after recovery due to residual myocardial fibrosis or scarring [7]. LGE with T2 abnormality on CMR suggests that myocardial edema is present and the myocarditis is in the acute inflammatory phase. Consequently, the extent of LGE can diminish after recovery [38]. In contrast, LGE without a T2 abnormality after recovery from myocarditis indicates myocardial scarring or fibrosis and is associated with a poorer prognosis [9, 39]. The prevalence of LGE in myocarditis patients other than COVID-19 dropped from 72 to 48% and that of a T2 abnormality decreased from 57 to 7% at 12 months follow-up in a previous study [38].

The time interval between a diagnosis of COVID-19 and CMR varied among the studies included in this meta-analysis. Nevertheless, CMR was performed within 22 weeks of COVID-19 diagnosis, a shorter interval than that reported in previous studies on non-COVID-19 myocarditis [38]. The pooled prevalence of CMR findings of acute myocarditis in recovered COVID-19 patients diagnosed with myocarditis (14.0%), elevated native T1 (26.3%), myocardial LGE (20.7%) and T2 abnormality (16.9%) was higher than that of myocardial LGE without T2 abnormality (4.0%), which indicates permanent myocardial scarring and is associated with a poor prognosis. A mid-wall septal pattern of LGE, a poor prognostic factor in non-COVID-19 myocarditis, has been reported in several studies [14, 16, 20, 28]. These results suggest that active myocardial inflammation persists in the early phase of recovery from COVID-19. Therefore, the results of large-scale, ongoing studies (C-MORE, CISCO-19 and COVID-HEART) with long-term follow-up may address whether these findings will disappear or remain as permanent myocardial fibrosis [40–42].

Myocarditis in athletes can be critical because athletes place themselves at a higher risk of sudden cardiac death or adverse cardiac events during strenuous exercise [25]. Currently, the consensus among experts does not recommend routine CMR for evaluating whether to allow athletes who recovered from COVID-19 to return to play [43–46]. Typically, CMR is not a first-line modality for evaluating patients with suspected myocardial injuries. Instead, CMR is performed after electrocardiography, cardiac biomarker analysis, or transthoracic echocardiography to provide a more advanced and comprehensive evaluation in patients with ongoing clinical concerns [43–46]. Although the prevalence of abnormal CMR findings was lower in athletes than in non-athletes in this meta-analysis, the prevalence of LGE without T2 abnormality was similar between the two groups. Moreover, the prevalence of pericardial LGE was higher in athletes than in non-athletes. Therefore, long-term follow-up studies with larger numbers of participants (athletes) who recovered from COVID-19 are necessary to determine the significance of LGE observed on CMR.

In this meta-analysis, we observed that patients with normal cardiac enzyme levels had less frequent CMR abnormalities than patients with unknown cardiac enzyme levels (59.4% vs. 35.9%). Although our meta-analysis could not include a subgroup analysis for patients with elevated cardiac enzymes, elevated troponin levels are well-established markers of myocardial injury. High troponin levels are associated with severe disease and a poor prognosis in COVID-19 patients [47, 48]. Elevated troponin levels in recovered COVID-19 patients suggests ongoing subclinical inflammation; however, it is uncertain whether normal cardiac enzyme levels indicate an absence of myocardial scars. CMR may provide risk stratification for patients who recovered from COVID-19.

Besides myocardial abnormality, ventricular systolic dysfunction and pericardial abnormalities have also been reported in recovered COVID-19 patients. RV systolic dysfunction is the most common cine abnormality in recovered COVID-19 patients and is associated with increased pulmonary vascular resistance [49], acute respiratory distress syndrome, and poor outcomes in patients with COVID-19 [50]. Although the prevalence of functional abnormalities is low relative to those observed for other CMR parameters, studies clarifying the mechanism underlying the restoration of cardiac function in these patients are needed. This meta-analysis revealed that pericardial effusion was frequently observed in recovered COVID-19 patients, whereas pericardial LGE was relatively rare. Pericarditis, pericardial effusion, and cardiac tamponade have occasionally been reported during the active phase of COVID-19 [51, 52]; however, the underlying mechanisms remain unclear. Inadequate immune response to COVID-19 may lead to slower clearance of the virus from the peri-myocardium, development of pericarditis secondary to myocardial inflammation, or pericardial effusion caused by generalized COVID-19-related multi-systemic inflammatory syndrome [13, 18, 21]. The outcome of this evidence is unknown; however, our study findings would support further study.

Comprehensive and definitive cardiac imaging guidelines for recovered COVID-19 patients, especially the non-athlete population, are lacking. Future large-scale, long-term studies may reveal the clinical significance of abnormal CMR findings. Based on our study and future studies, appropriate surveillance guidelines for using CMR and other cardiac imaging modalities in recovered COVID-19 patients should be established.

### Limitations

Our study has several limitations. First, the subgroup of patients with elevated cardiac enzyme levels could not be analyzed due to the small number of studies and patients. Second, an analysis of ventricular systolic dysfunction in the subgroup of patients with normal cardiac enzyme levels was not conducted due to the small number of patients with ventricular systolic dysfunction. Third, certain data necessary for subgroup analysis, such as the presence of cardiac symptoms or underlying cardiac disease, or abnormalities revealed on electrocardiography or echocardiography, could not be extracted. Lastly, CMR scans were performed within 22 weeks of COVID-19 recovery, and longer-term studies are needed to determine the clinical significance of these findings.

## Conclusions

Nearly half of those recovering from COVID-19 exhibit one or more abnormal CMR findings. The prevalence of abnormal CMR findings was lower in athletes and patients with normal cardiac enzyme levels than in non-athletes and patients with undetermined cardiac enzyme levels. We propose that comprehensive surveillance with CMR could help stratify the risks of cardiovascular complications in recovered COVID-19 patients.

## Supplementary Information

**Additional file 1.Supplementary Table 1.** Quality assessment of the included studies. **Supplemental Appendix 1.** Search terms used in PubMed, the Cochrane library and EMBASE. **Supplementary Figure 1.** Funnel plots to detect publication bias.

## Data Availability

The dataset analyzed during the current study are available from the corresponding author on reasonable request.

## Abbreviations

CI: Confidence interval
CMR: Cardiovascular magnetic resonance
COVID-19: Coronavirus disease 2019
CRP: C reactive protein
ECG: Electrocardiogram
ECV: Extracellular volume
LGE: Late gadolinium enhancement
LV: Left ventricle/left ventricular
LVEF: Left ventricular ejection fraction
RV: Right ventricle/right ventricular
RVEF: Right ventricular ejection fraction
T2w: T2-weighted
