# Supplementary material for: Prevalence of abnormal cardiovascular magnetic resonance findings in recovered patients from COVID-19: a systematic review and meta-analysis
Source: J Cardiovasc Magn Reson. 2021 Sep 3;23:100. doi: 10.1186/s12968-021-00792-7 (PMC8414035; doi:10.1186/s12968-021-00792-7)
Supplement: Supplementary file 1 — Additional file 1.Supplementary Table 1. Quality assessment of the included studies. Supplemental Appendix 1. Search terms used in PubMed, the Cochrane library and EMBASE. Supplementary Figure 1. Funnel plots to detect publication bias. [file 12968_2021_792_MOESM1_ESM.docx]

**Supplementary Material**

**Supplementary Table 1. Quality assessment of the included studies.**

| First author | Selection | | | | Comparability | Outcome | | | Total score |
| --- | --- | --- | --- | --- | --- | --- | --- | --- | --- |
|  | Representativeness of the exposed cohort | Selection of the non-exposed cohort | Ascertainment of exposure | Demonstration that the outcome of interest was not present at the start of the study | Comparability of cohorts based on the design or analysis | Assessment of outcome | Was follow-up long enough for outcomes to occur? | Adequacy of the follow-up of cohorts |  |
| Ng et al. (2020) | 1 | 1 | 1 | 0 | 0 | 1 | 1 | 1 | 6 |
| Huang et al. (2020) | 1 | 1 | 1 | 0 | 0 | 1 | 1 | 1 | 6 |
| Rajpal et al. (2020) | 1 | 1 | 1 | 0 | 0 | 1 | 1 | 1 | 6 |
| Clark et al. (2021) | 1 | 1 | 1 | 0 | 0 | 1 | 1 | 1 | 6 |
| Knight et al. (2020) | 1 | 0 | 1 | 1 | 0 | 1 | 1 | 1 | 6 |
| Puntmann et al. (2020) | 1 | 1 | 1 | 1 | 0 | 1 | 1 | 1 | 7 |
| Eiros et al. (2020) | 1 | 1 | 1 | 1 | 0 | 1 | 1 | 1 | 7 |
| Vago et al. (2020) | 1 | 1 | 1 | 0 | 0 | 1 | 1 | 1 | 6 |
| Brito et al. (2020) | 1 | 1 | 1 | 0 | 0 | 1 | 1 | 1 | 6 |
| Malek et al (2021) | 1 | 0 | 1 | 0 | 0 | 1 | 1 | 1 | 5 |
| Li et al (2021) | 1 | 1 | 1 | 1 | 0 | 1 | 1 | 1 | 7 |
| Starekova et al (2021) | 1 | 0 | 1 | 0 | 0 | 1 | 1 | 1 | 5 |
| Wang et al (2021) | 1 | 1 | 1 | 1 | 0 | 1 | 1 | 1 | 7 |
| Pan et al (2021) | 1 | 1 | 1 | 1 | 0 | 1 | 1 | 1 | 7 |
| Zhou et al (2021) | 1 | 0 | 1 | 1 | 0 | 1 | 1 | 1 | 6 |
| Kotecha et al (2021) | 1 | 1 | 1 | 1 | 0 | 1 | 1 | 1 | 7 |

**Supplemental Appendix 1. Search terms used in PubMed, the Cochrane library and EMBASE**

1. Search terms for PubMed and the Cochrane library:

Myocardium[MeSH Terms] OR Heart[MeSH Terms] OR Heart Ventricles[MeSH Terms] OR Myocarditis[MeSH Terms] OR Heart or myocardium or myocardial or cardiac or ventricle or ventricular or myocarditis or (myocardial injury)

AND ((("COVID-19"[Supplementary Concept] OR "Coronavirus"[MeSH Terms]) OR "severe acute respiratory syndrome coronavirus 2"[Supplementary Concept]) OR ((((("Coronavirus"[MeSH Terms] OR "Coronavirus"[All Fields]) OR "coronaviruses"[All Fields]) OR ((((((("COVID-19"[All Fields] OR "covid 2019"[All Fields]) OR "severe acute respiratory syndrome coronavirus 2"[Supplementary Concept]) OR "severe acute respiratory syndrome coronavirus 2"[All Fields]) OR "2019 ncov"[All Fields]) OR "sars cov 2"[All Fields]) OR "2019ncov"[All Fields]) OR (("wuhan"[All Fields] AND ("Coronavirus"[MeSH Terms] OR "Coronavirus"[All Fields])) AND (2019/12/1:2019/12/31[Date - Publication] OR 2020/1/1:2020/12/31[Date - Publication])))) OR (("severe acute respiratory syndrome coronavirus 2"[Supplementary Concept] OR "severe acute respiratory syndrome coronavirus 2"[All Fields]) OR "2019 ncov"[All Fields])) OR (("severe acute respiratory syndrome coronavirus 2"[Supplementary Concept] OR "severe acute respiratory syndrome coronavirus 2"[All Fields]) OR "sars cov 2"[All Fields])))

AND Magnetic Resonance Imaging"[Mesh] OR "Magnetic Resonance Imaging, Cine"[Mesh] or magnetic resonance or MR or CMR or MRI or cmri

2) Search terms for EMBASE:

(('heart'/exp OR 'cardiac muscle'/exp OR 'myocarditis'/exp) OR (heart OR myocardium OR myocardial OR cardiac OR ventricle OR ventricular OR myocarditis OR (myocardial AND injury))) AND (('coronavirus infection'/exp OR 'coronavirus disease 2019'/exp) OR (coronavirus OR 'covid 19' OR '2019 ncov' OR 'sars cov 2')) AND (('cardiovascular magnetic resonance'/exp OR 'cine magnetic resonance imaging'/exp) OR (magnetic AND resonance OR mr OR cmr OR mri OR cmri))

**Supplementary Figure 1. Funnel plots to detect publication bias.**

Each circle represents a study included in the analysis. Probability values of asymmetry from the Egger tests are shown.

1. Prevalence of total abnormal CMR findings


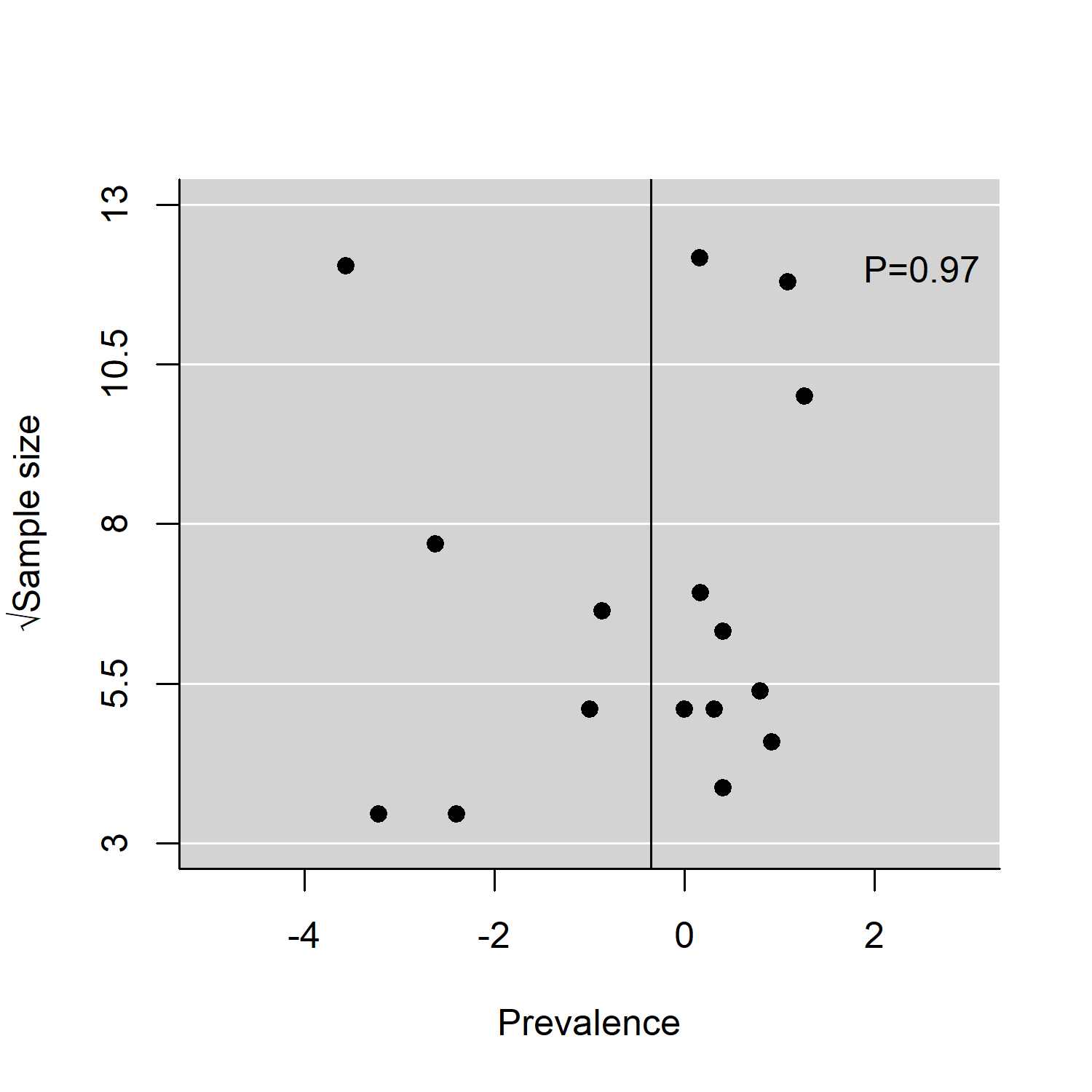


1. Prevalence of the diagnosis of myocarditis on CMR


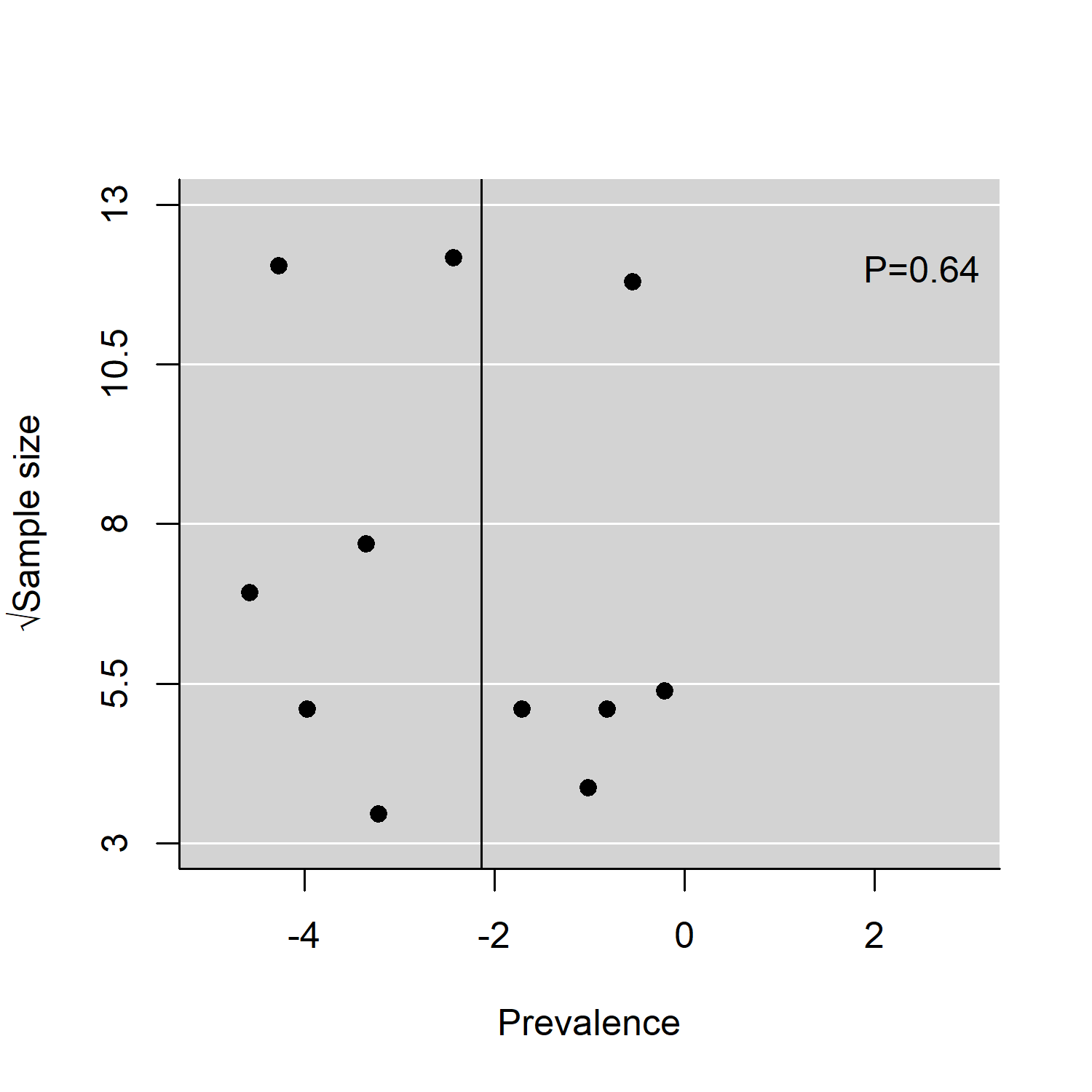


1. Prevalence of myocardial LGE


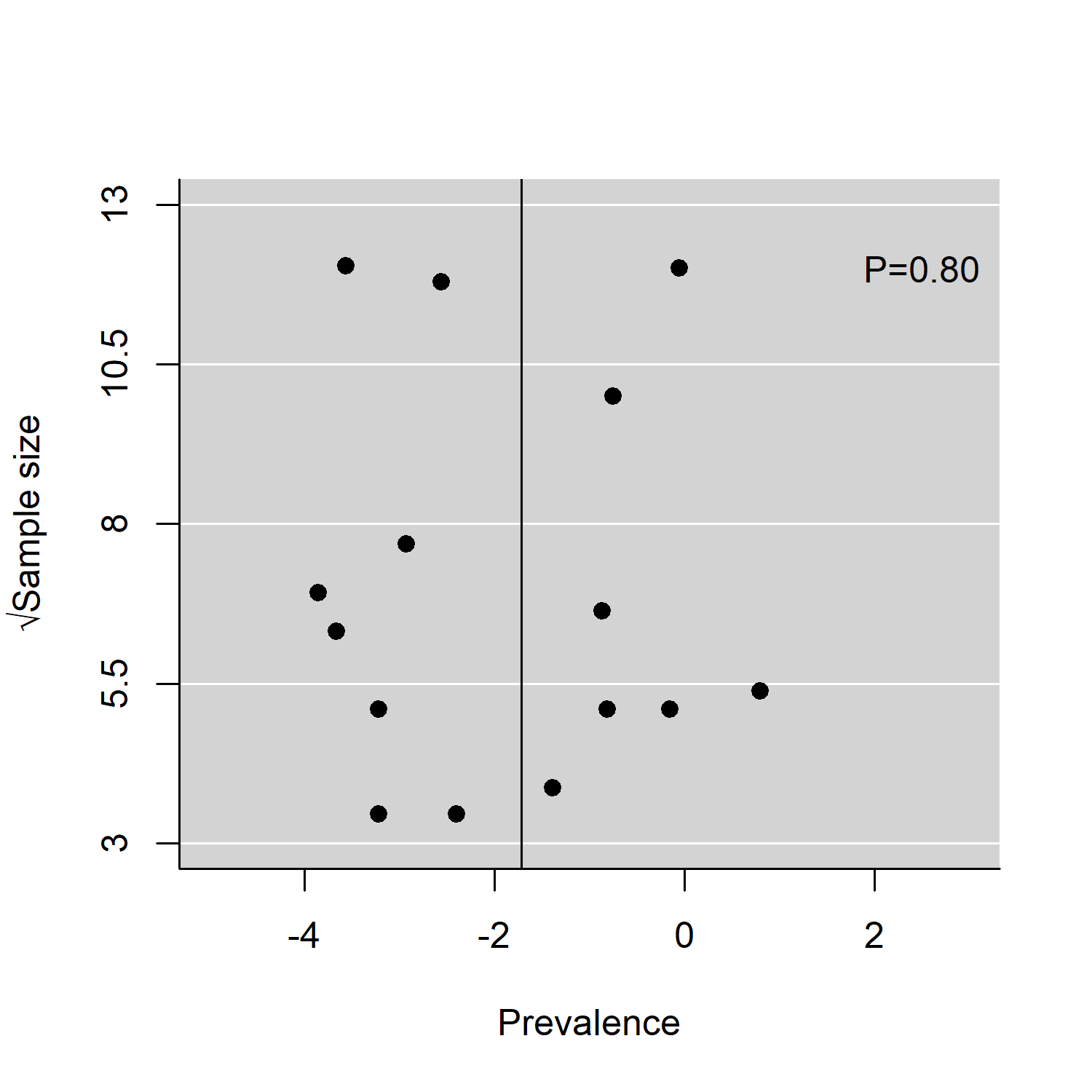


1. Prevalence of pericardial LGE


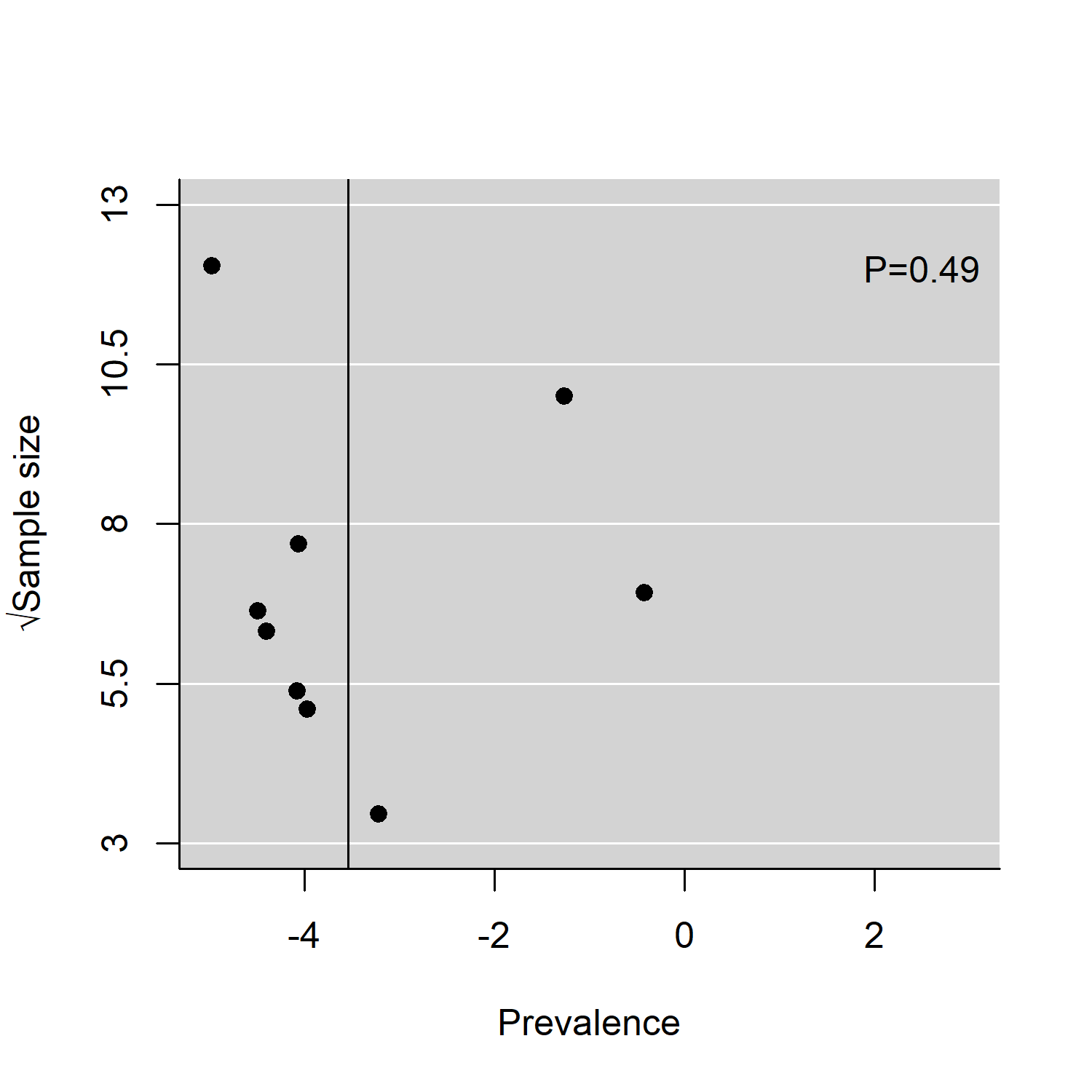


1. Prevalence of increased T1 value on T1 map
2.
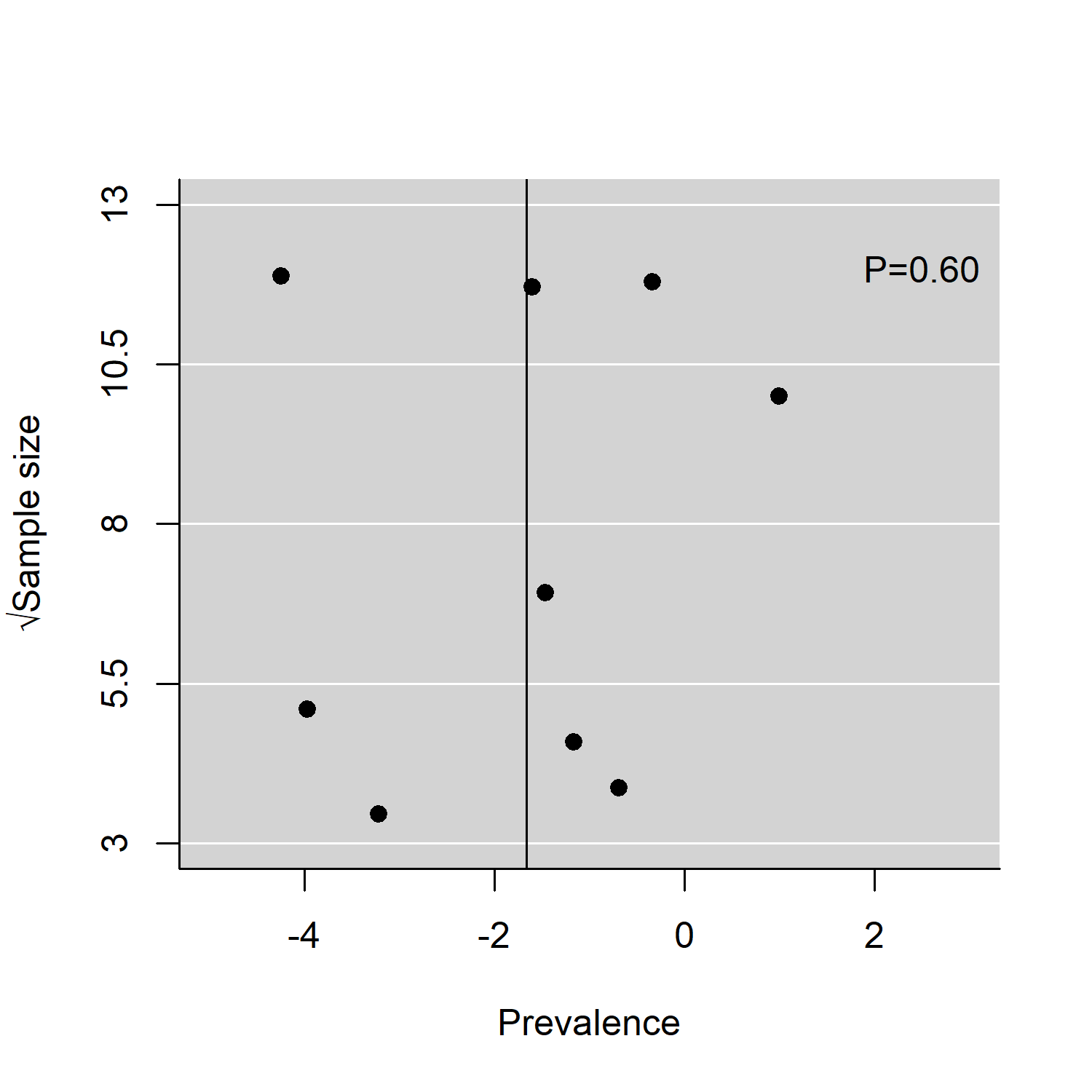

3. Prevalence of T2 abnormality


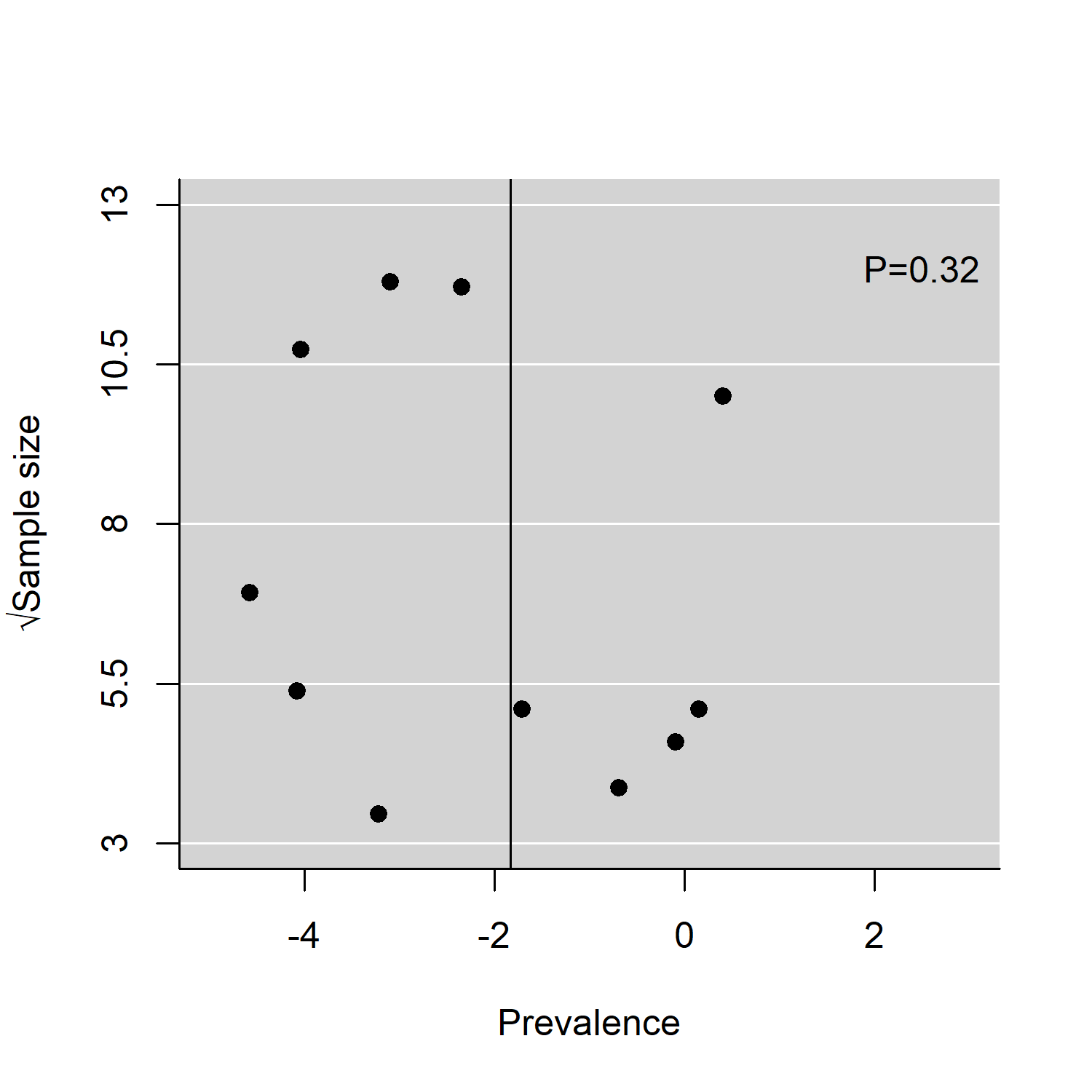


1. Prevalence of LGE without T2 abnormality


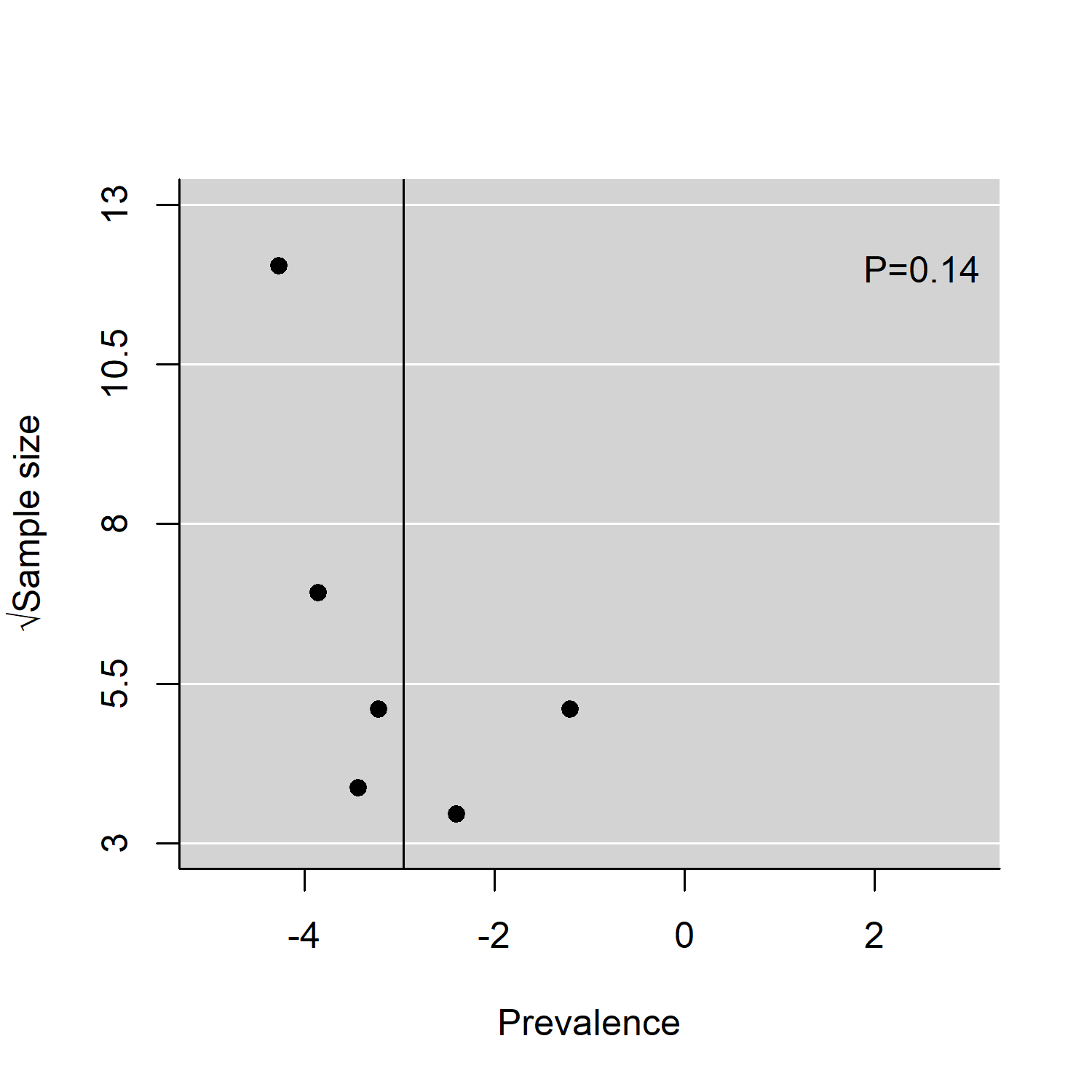


1. Prevalence of abnormal T2 without LGE


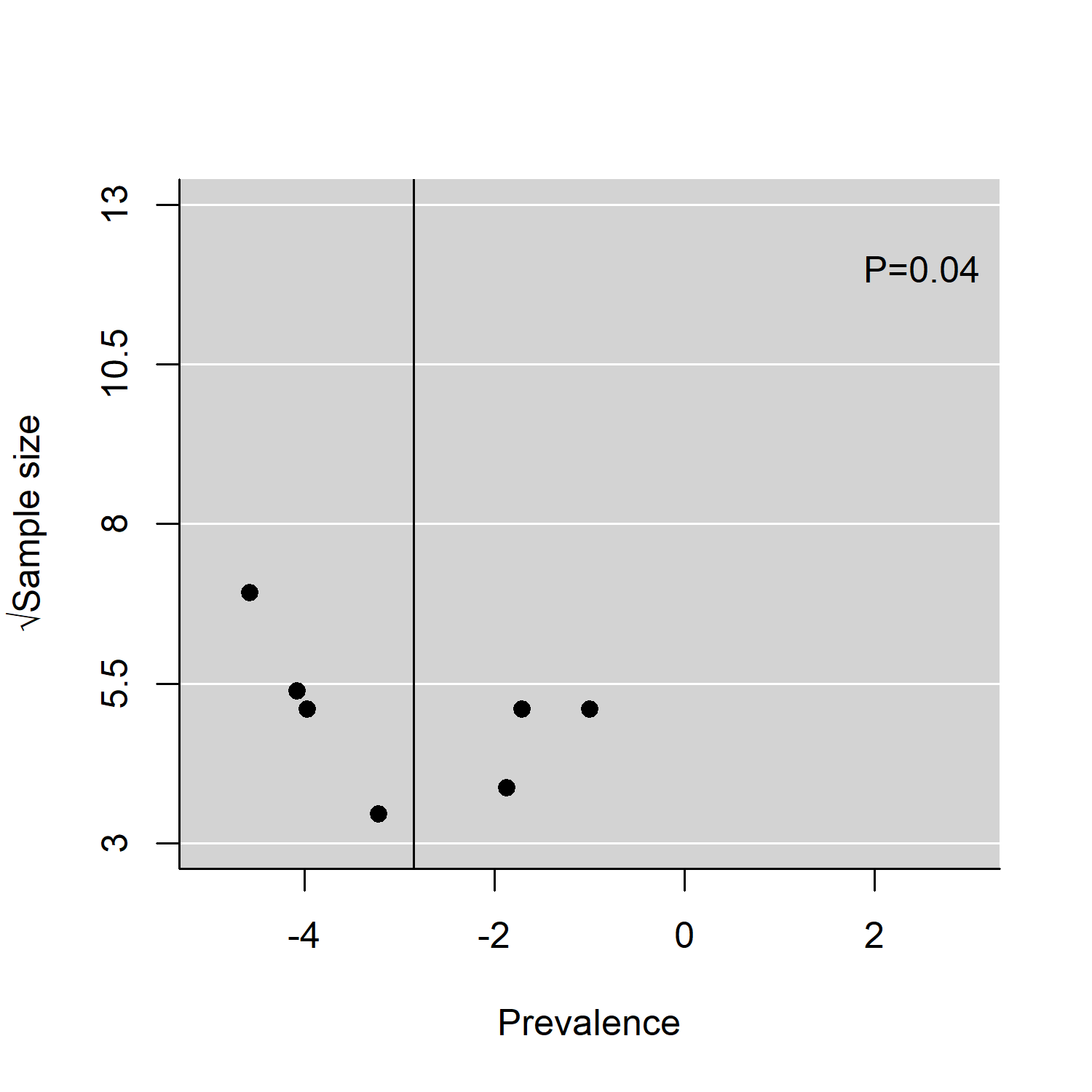


1. Prevalence of ventricular dysfunction


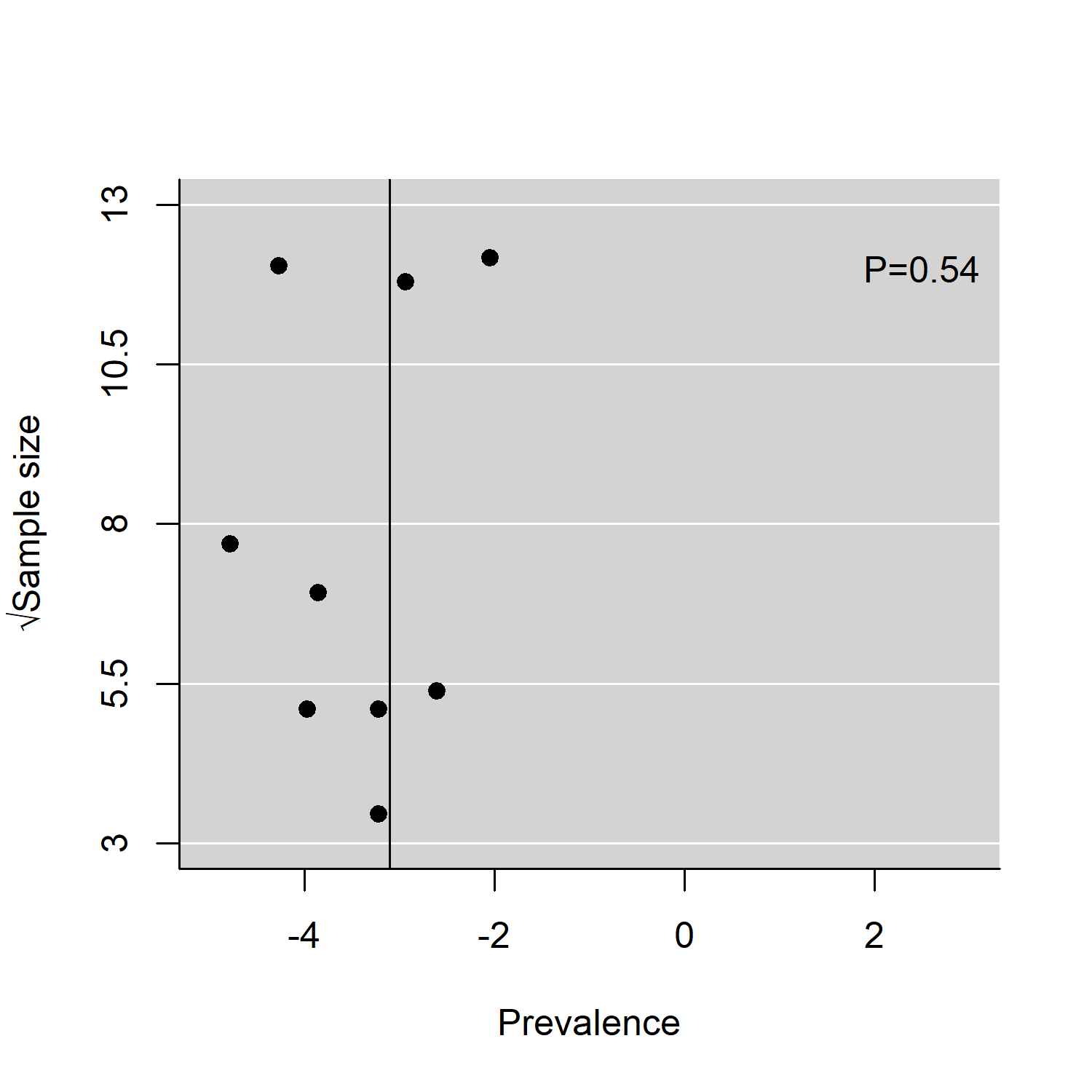


1. Prevalence of pericardial effusion


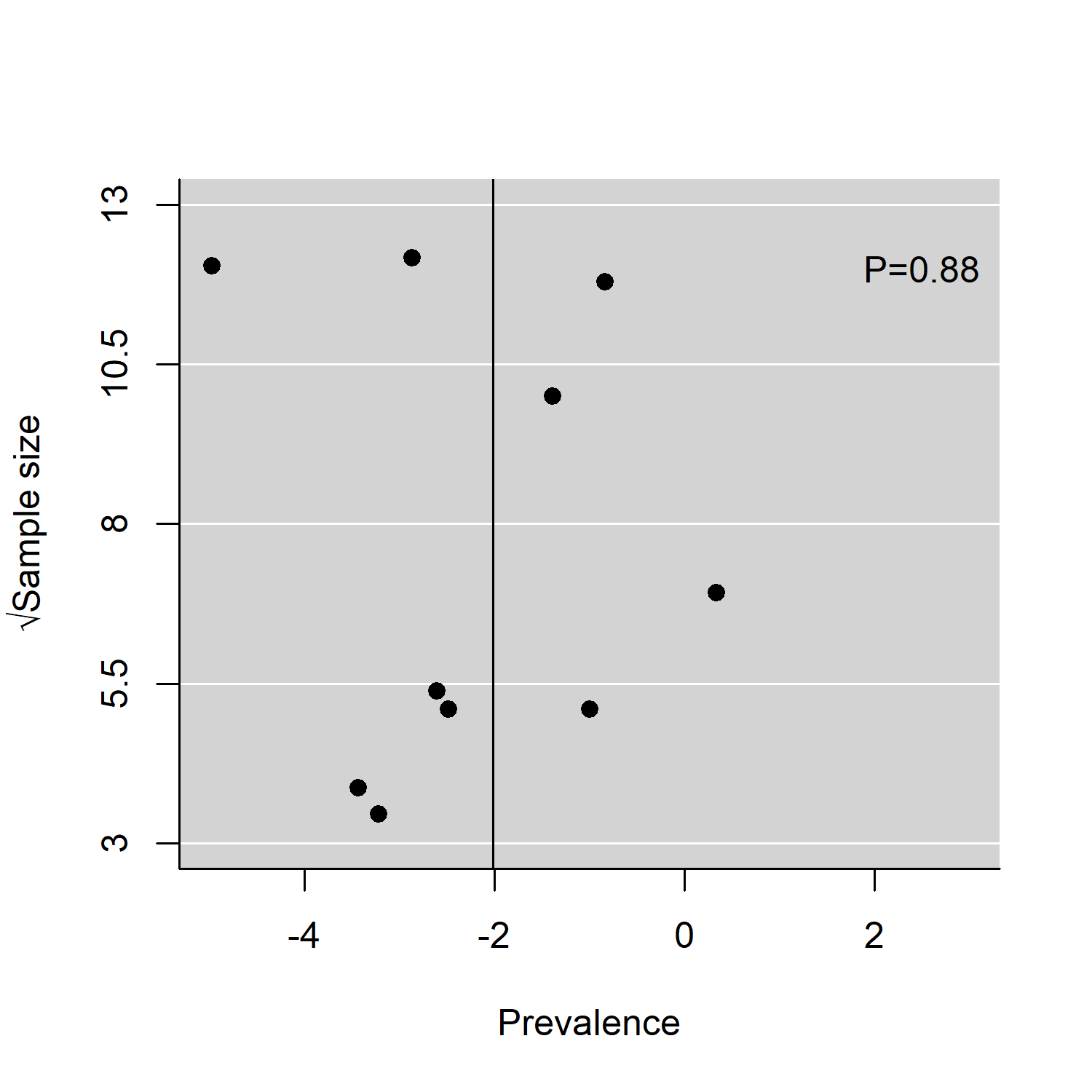


CMR: cardiac magnetic resonance imaging, LGE: late gadolinium enhancement.
